# The reverse transcription signature of *N*-1-methyladenosine in RNA-Seq is sequence dependent

**DOI:** 10.1093/nar/gkv895

**Published:** 2015-09-13

**Authors:** Ralf Hauenschild, Lyudmil Tserovski, Katharina Schmid, Kathrin Thüring, Marie-Luise Winz, Sunny Sharma, Karl-Dieter Entian, Ludivine Wacheul, Denis L. J. Lafontaine, James Anderson, Juan Alfonzo, Andreas Hildebrandt, Andres Jäschke, Yuri Motorin, Mark Helm

**Affiliations:** 1 Institute of Pharmacy and Biochemistry, Johannes Gutenberg University Mainz, Staudingerweg 5, 55128 Mainz, Germany; 2 Institute of Pharmacy and Molecular Biotechnology (IPMB), Heidelberg University, Im Neuenheimer Feld 364, 69120 Heidelberg, Germany; 3 Institute of Molecular Biosciences: Goethe University Frankfurt, Max-von-Laue Street 9, 60438 Frankfurt/M, Germany; 4 RNA Molecular Biology, Université Libre de Bruxelles, Rue Profs Jeener & Brachet, 12, B-6041 Charleroi-Gosselies, Belgium; 5 Department of Biological Sciences, Marquette University, 53201-1881, Milwaukee, WI, USA; 6 Department of Microbiology, The Ohio State University, 43210, Columbus, OH, USA; 7 Institute for Computer Sciences, Johannes Gutenberg University Mainz, Staudingerweg 9, 55128 Mainz, Germany; 8 IMoPA UMR7365 CNRS-UL, BioPole de l'Université de Lorraine, 9 avenue de la Foret de Haye, 54505 Vandoeuvre-les-Nancy, France

## Abstract

The combination of Reverse Transcription (RT) and high-throughput sequencing has emerged as a powerful combination to detect modified nucleotides in RNA via analysis of either abortive RT-products or of the incorporation of mismatched dNTPs into cDNA. Here we simultaneously analyze both parameters in detail with respect to the occurrence of *N*-1-methyladenosine (m^1^A) in the template RNA. This naturally occurring modification is associated with structural effects, but it is also known as a mediator of antibiotic resistance in ribosomal RNA. In structural probing experiments with dimethylsulfate, m^1^A is routinely detected by RT-arrest. A specifically developed RNA-Seq protocol was tailored to the simultaneous analysis of RT-arrest and misincorporation patterns. By application to a variety of native and synthetic RNA preparations, we found a characteristic signature of m^1^A, which, in addition to an arrest rate, features misincorporation as a significant component. Detailed analysis suggests that the signature depends on RNA structure and on the nature of the nucleotide 3′ of m^1^A in the template RNA, meaning it is sequence dependent. The RT-signature of m^1^A was used for inspection and confirmation of suspected modification sites and resulted in the identification of hitherto unknown m^1^A residues in trypanosomal tRNA.

## INTRODUCTION

1-methyladenosine (m^1^A) is an RNA modification originating essentially from two different reaction types, one catalyzed by enzymes and the other the result of the reaction of RNA with certain alkylating agents. Correspondingly, the relevance of this modification in RNA-related research is essentially two-fold. On one hand, dimethylsulfate (DMS) is a popular chemical probe of RNA structure in solution; reactivity toward DMS is interpreted as accessibility of the corresponding nitrogen or nucleobase to solvent, and hence a lack of structural involvement. The *N1* of adenosine is not the only RNA nucleophile to react with DMS (1), as e.g. the *N3* of cytidines and the *N7* of guanosines are also probed by this reagent. For the latter two, the resulting chemically modified nucleosides m^3^C and m^7^G can be revealed by further chemical treatments leading to chain scission at the modified sites. Since such a treatment has not been developed for m^1^A, it has traditionally been detected by primer elongation arrest (2,3). The underlying logic is that chemical alterations blocking the Watson–Crick face should act as an RT-roadblock, as such impair the incorporation of the complementary nucleotide into the cDNA by the reverse transcriptase enzyme, and cause the latter to stall. A typical result of structural probing experiments is thus an arrest signal at the position of the last nucleotide upstream or the 5′-adjacent nucleotide of the cDNA (corresponding to the 3′-nucleotide of m^1^A on the RNA template). While the traditional method for detecting such RT-arrest signals involves the resolution of labeled primer extension products by polyacrylamide gelelectrophoresis (PAGE) or capillary electrophoresis, recent developments in structural probing make use of the power of deep sequencing methods (4). Of note, in the entire field, the tacit assumption for decades has been that m^1^A is quantitative in its RT-arrest capacity, i.e. structural probing experiments were interpreted as if every encounter of an m^1^A by an RT enzyme led to abortion of primer elongation. On the other hand, m^1^A is also a prominent and frequently occurring member of the growing family of 150 or so chemically different naturally occurring RNA modifications. It is typically found at position 58 of many eukaryotic and archaeal tRNAs (5), as well as in eukaryotic (6) and bacterial (7–9) rRNA. Further occurrences are known from position 9 of metazoan mitochondrial tRNAs (9–11), and as a mediator of antibiotic resistance in rRNA of *Streptomyces pactum* (12).

Interestingly, several recent papers, including applications of different RNA-Seq protocols have created data containing mismatched nucleoside signals at sites known or postulated to contain m^1^A (13,14). This strongly suggests that reverse transcriptase is capable of reading through this altered Watson–Crick face, thereby incorporating non-matching nucleotides in the process and leaving unobtrusive traces of the m^1^A modification in cDNA data. Such behavior is known from DNA polymerase bypassing sites of DNA lesion (15). In a comprehensive investigation of misincorporation caused by various RNA modifications, Ryvkin *et al*. have recently reported a common misincorporation pattern for adenosine modifications (16). However, the protocols for library preparation in the reported RNA-Seq approach were unsuited to detect the abortive cDNA products described above in the structural probing context. Failure to detect RT-arrest signals may originate from details of the library preparation protocols, for example when both primer binding sites are introduced via ligation on the RNA level. For the detection of RT-arrest signals e.g. in tRNA (17), the second primer binding site must be introduced at the level of cDNA.

**Figure 1.**
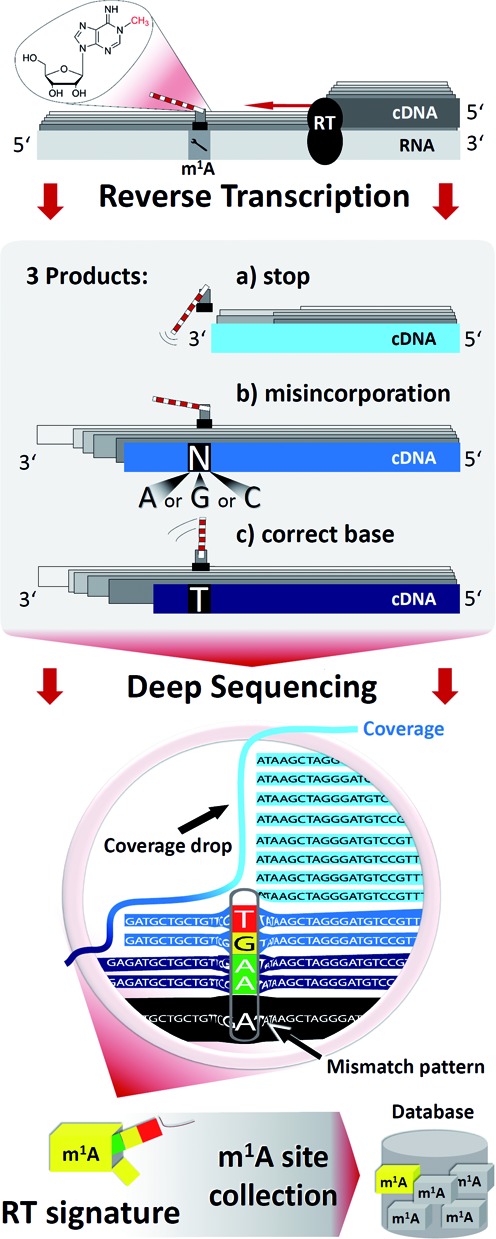
Principle of generation and analysis of RNA-Seq data for the detection of m^1^A residues.

Here, we use a library preparation protocol suitable for the detection of both, abortive cDNA and misincorporation (Figure 1). Application to RNA preparations containing known or suspected m^1^A sites revealed an RT-signature left by m^1^A residues which includes characteristic misincorporation patterns as well as typical RT-arrest rates. Most interestingly, we find a dependence on the type of the m^1^A-preceding nucleotide in the RNA template (i.e. to the 3′ of m^1^A), whose nature correlates with misincorporation patterns. These findings have important bearings for both areas: in structural probing, proper interpretation of RT-arrest assays of DMS treated RNA should include the notion of incomplete detection as well as a sequence context around the adenosine residue under investigation.

## MATERIALS AND METHODS

Unless otherwise specified, all synthetic nucleic acids were from IBA (Göttingen, Germany). Details including sequence information are given in Supplementary Table S1. Yeast rRNA was prepared as described in (6), yeast tRNA as described in (18). *S. pactum* DSM40530 (DSMZ, Braunschweig, Germany) was cultivated as recommended for liquid media growth (19) with slight modifications. Total bacterial RNA was extracted with TRIzol^®^ Reagent (life technologies, Thermo Scientific, Dreieich, Germany) according to the manufacturer's protocol.

### Library preparation protocol

This protocol was slightly varied from a previously published version (18, 20) as follows: the RNA adapter of 5 random (N) nucleotides + 1 constant cytidine (C) at the 3′ end was replaced by a 9 N + 1 C Illumina p5 template sequence. The custom sequence of the cDNA adapter was substituted with an Illumina p7 template sequence. Instead of 6 nt at each 5′ end of primers as barcode, full length Illumina compatible (derived from Nextera 2 platform) primers with dual barcodes N501-N508 and N701-N7012 were used. TrueSeq DNA amplicon library preparation for introducing Illumina compatible sequences before sequencing was not required.

#### Fragmentation

Total or ribosomal RNA was fragmented in a volume of 10 μl containing 10 mM ZnCl_2_, and 100 mM Tris–HCl, pH 7.4, at 90°C for 5 min. The reaction was stopped by addition of ethylenediaminetetraacetic acid (EDTA) to a final concentration of 50 mM. Next, the RNA was size separated by 10% denaturating PAGE and bands of size 50–150 nt were excised, eluted in 0.3 M ammonium acetate and ethanol precipitated.

#### Dephosphorylation

RNA (about 0.5 μg) was then dephosphorylated on both extremities in dephosphorylation mixture (10 μl total) consisting of 100 mM Tris–HCl, pH 7.4, 20 mM MgCl_2_, 0.1 mg/ml BSA, 100 mM 2-mercaptoethanol, and 0.5 U FastAP (Thermo Scientific) at 37°C for 30 min. Prior to addition of enzyme, RNA was denatured at 90°C for 30 s, then chilled on ice (in the following, this will be referred to as ‘heat denaturation’). After 30 min reaction time, RNA was again heat denatured and an equal amount of enzyme was added to perform a second cycle of dephosphorylation.

#### 3′-adapter ligation

Next, an adapter was ligated to the 3′-end of dephosphorylated RNA. To this end, dephosphorylated RNA was complemented to yield a ligation mixture (final volume 20 μl) and to perform ligation as described in (18). Here, RAdapter (IBA, Goettingen, Germany; see Supplementary Table S1 for Sequence) was used in a concentration of 5 μM. After the reaction the enzymes were inactivated at 75°C for 15 min.

#### Removal of excess adapters

Before the reverse transcription step, the excess of RAdapter was removed. To this end, the mixture was heat denatured. Then 20 U of 5′–Deadenylase (New England Biolabs, Frankfurt, Germany) were added to the ligation mix, followed by incubation at 30°C for 30 min. After a second heat-denaturation an equal amount of enzyme was added and the reaction was repeated. Next, the single stranded RAdapter (now completely monophosphorylated) was digested by adding 10 U of Lambda exonuclease (Thermo Scientific, Dreieich, Germany) to the reaction mixture and incubating at 37°C for 30 min. After heat denaturation an equal amount of enzyme was added to repeat the reaction. Finally, both enzymes were heat-inactivated at 80°C for 15 min, after which RNA was ethanol precipitated with the addition of 1μl glycogen (Thermo Scientific, Dreieich, Germany) per sample.

#### Reverse transcription

Composition of the reverse transcription mixture (here, 40 μl) was previously described (18). Here, the pellet was first redissolved in 32 μl RTPrimer (IBA, Goettingen, Germany; see Supplementary Table S1 for sequence) in a final concentration of 5 μM in FS Buffer (Life Technologies) and heat denatured at 80°C for 10 min, then chilled on ice. Then, dNTP mix BSA, dithiothreitol and SuperScript III reverse transcriptase (10 U/μl, Life Technologies) were added. Reactions were performed at 50°C for 1 h and no heat inactivation was performed.

#### Removal of excess primers and dNTPs

Next, the RTPrimer was digested. To this end, 20 U of Lambda exonuclease were added to the reverse transcription mix, and incubated at 37°C for 30 min. The reaction was repeated by addition of an equal amount of enzyme, without prior heat denaturation, to avoid denaturation of RNA:DNA hybrids. Following this, 80 U of single-strand specific Exonuclease I (Thermo Scientific) were added and incubated at 37°C for 30 min. Again, the reaction was repeated by addition of an equal amount of enzyme, without prior heat-denaturation. Finally, all enzymes were heat-inactivated at 80°C for 15 min. After that, dNTPs were dephosphorylated. For this, 4 U of FastAP thermosensitive alkaline phosphatase were added to the mixture and incubated at 37°C for 30 min. The reaction was repeated upon heat-denaturation. Finally, RNA was hydrolyzed as described (18). The reaction was stopped by neutralizing with an equal amount of acetic acid and precipitating with ethanol.

#### 3′-tailing and ligation of cDNA

The obtained cDNA was reacted with TdT (Thermo Scientific, Dreieich, Germany) as published in a volume of 10 μl. Oligocytidine overhangs were generated using cytidine triphosphate (CTP) under optimized conditions affording >90% addition of three cytidines (18). For the ligation of the second adapter the TdT mixture was complemented to yield a final ligation mixture consisting of 50 mM Tris–HCl, pH 7.4, 20 mM MgCl_2_, 1.25 μM DAnchor (DAnchorA annealed to DAnchorB, IBA, Goettingen, Germany; see Supplementary Table S1 for sequence), 10μM ATP and 1.5 Weiss U/μl T4 DNA ligase (Thermo Scientific) in a total volume of 40 μl. Reaction was performed and ligation products purified as described (18).

#### PCR amplification and barcoding

Each sample was finally polymerase chain reaction (PCR) amplified using the respective barcoded P7 and P5 primers (IBA, Goettingen, Germany, see Supplementary Table S1). PCR products were size-separated by 10% denaturing PAGE and regions of interest (above 150, which is the size of adapter dimers and below 300, which is the maximum size of PCR amplicons) were excised, DNA was eluted in 0.3 M ammonium acetate and ethanol precipitated. The resuspended DNA was then sequenced on the MiSeq platform (see Supplementary Table S2 for details).

### Deep-Seq data processing and mapping

The sequence libraries specified in Supplementary Table S2 (end-types, lengths, platform) were processed in a custom bioinformatic pipeline. Corresponding to the library preparation settings, a Python based workflow accommodated demultiplexing, removal of primers, adapters, barcodes and ligation-assistance overhangs. Mapping was performed using Bowtie2 with solely tRNA or rRNA references obtained from MODOMICS (9) for the corresponding organism. Alignment mode was set to global (end-to-end, no soft-clipping) with one mismatch tolerated in the seed of 6 nt. Splicing was not part of the mapping strategy. Mapping to all references simultaneously, only one (*k* = 1) alignment declared as valid by Bowtie2 was reported for each read.

### Signature extraction

Mapping was followed by format conversion using SAMtools. From SAM files, sorted and indexed BAM files were generated, which were translated to Pileup format. An additional conversion lead to a custom tab-separated text file format, termed *Profile* (details in Supplementary Table S3), providing all parameters of relevance for inspection of modification candidates. Herein, for each reference position the listed properties include coverage *c*, arrest rate *a*, mismatch content *m* as well as the counts for each base type. All presented RT signatures were compiled manually during visual inspection of the mapping results. Database entries of m^1^A sites listed in MODOMICS were retrieved and confirmed by evaluation of arrest rate characteristics and mismatch patterns. The extracted signatures were complemented by those of m^1^As from homologous identification performed via ClustalW2 sequence alignments of related organisms. Identification was performed by isolated visual inspection. By manual selection, positional shifts of m^1^A_58_ to e.g. positions 57 or 59 due to variable loops were correctly recognized and from all sites listed in Modomics, those could be determined that obtained a signature projected by our approach.

### Supervised prediction

The uniqueness of m^1^A's RT signature was evaluated by supervised prediction, i.e. machine learning mediated detection of known m^1^A instances within a pool of non-methylated adenosine sites with m^1^A-resembling or differing sequencing profiles. The general workflow is shown in Supplementary Figure S6. Mean prediction performances (sensitivity, specificity) were calculated from 10 repetitions of a five-fold stratified cross-validation, training and testing a Random Forest (RF) *R* package implementation (21). The training sets contained equal amounts of instances of both classes. Attributes used for classification input were its arrest rate *a*, relative mismatch content *m*, relative mismatch composition values (G, T and C content), *m*/*a* and the fold change of a w.r.t. the mean *a* within the site's −5 and +5 bp neighborhood, termed context sensitive arrest rate (CSA). The input format of training material is detailed in Supplementary Table S4. In the first input setting, (i), all 45 m^1^A signatures from tRNA (already averaged for isotypes), rRNA and synthetic oligoribonucleotides were merged with 45 random non-m^1^A instances. The isotype averaging ensures that for any distribution of the data into training and testing sets, the classifier is facing unseen data in a test run. From (i), setting (ii) was derived, which allowed only non-m^1^A of a minimum m^1^A signature resemblance w.r.t. at least one of the thresholds a ≥ 0.2, m ≥ 0.2 or at least two mismatch type with ≥ 0.1 share of an m ≥ 0.1. Setting (iii) corresponded to (i) except that the training set was generated from tRNA instances (*Saccharomyces cerevisiae* cytosolic and *Homo sapiens* mitochondrial) only, while the test set contained rRNA sites (*S. cerevisiae*, *S. pactum*) exclusively. To demonstrate the advantage of our prediction model, we compared the supervised prediction power of the RF with that of a basic k-nearest neighbor (kNN) classifier (Supplementary Figure S7).

### LC-MS/MS analysis

#### HPLC-DAD-MS/MS analysis

##### Isolation of single tRNA species from *Trypanosoma brucei*

Single tRNA species were isolated from *Trypanosoma brucei* total RNA (22) by hybridization with complementary, biotinylated DNA-oligonucleotides followed by immobilization on streptavidin-coated magnetic beads (Dynabeads^®^ MyOne™ Streptavidin T1, Life Technologies, Darmstadt, Germany). Target tRNA was tRNA^Arg(UCG)^ (oligonucleotide 4309, sequence: biotin-CGGCAGGACTCGAACCTGCAACCCTCA). The hybridization step was performed in 5× SSC buffer (20×: 3 M NaCl, 300 mM trisodium citrate, pH 7.0) using 100 pmol biotinylated oligonucleotide and 150 μg total RNA per 25 μl beads. Samples were denatured at 90°C for 3 min and subsequently hybridized at 65°C for 10 min and cooled to room temperature. Dynabeads^®^ were washed three times using Binding and Washing buffer (5 mM Tris–HCl (pH 7.5), 0.5 mM EDTA, 1 M NaCl) according to the manual and then equilibrated once in 5× SSC buffer before adding the hybridized samples. Immobilization of the hybrid was performed at 25°C under shaking for 30 min. Subsequently, the supernatant containing non-target tRNAs was removed and the beads were washed once in 1× SSC buffer and three times in 0.1× SSC buffer. Finally, the beads were resuspended in MilliQ water and heated to 75°C for 3 min to elute the target tRNA. To exclude the presence of remaining DNA-oligonucleotide and non-target RNAs, the eluted RNA was further purified by 10% denaturing polyacrylamide gel electrophoresis and ethanol precipitation.

##### Sample preparation

Prior to LC-MS/MS analysis, RNA samples were digested into nucleosides according to the following protocol: samples were incubated in presence of 1/10 volume of 10× nuclease P1 buffer (0.2 M ammonium acetate pH 5.0, ZnCl_2_ 0.2 mM), 0.3 U nuclease P1 (Sigma Aldrich, Munich, Germany) and 0.1 U snake venom phosphodiesterase (Worthington, Lakewood, USA) at 37°C for 2 h. Next, 1/10 volume of 10× fast alkaline phosphatase buffer (Fermentas, St Leon-Roth, Germany) and 1 U fast alkaline phosphatase (Fermentas, St Leon-Roth, Germany) were added, and samples were incubated for additional 60 min at 37°C. After digestion, 1/10 volume of ^13^C-labeled total RNA (*S. cerevisiae*, 10 ng/μl), prepared as described in (23), was added as internal standard for m^1^A quantification.

##### HPLC parameters

The calibration solutions and digested RNA samples were analyzed on an Agilent 1260 HPLC series equipped with a diode array detector (DAD) and a triple quadrupole mass spectrometer (Agilent 6460). A Synergy Fusion RP column (4 μm particle size, 80 Å pore size, 250 mm length, 2 mm inner diameter) from Phenomenex (Aschaffenburg, Germany) was used at 35°C column temperature. The solvents consisted of 5 mM ammonium acetate buffer adjusted to pH 5.3 using acetic acid (solvent A) and pure acetonitrile (solvent B). The elution was performed at a flow rate of 0.35 ml/min using a linear gradient from 0 to 8% solvent B at 10 min, 40% solvent B at 20 min and 0% solvent B at 23 min. For additional 7 min, the column was rinsed with 100% solvent A to restore the initial conditions.

##### MS parameters

Prior to entering the mass spectrometer, the effluent from the column was measured photometrically at 254 nm by the DAD. The triple quadruple mass spectrometer, equipped with an electrospray ion source (Agilent Jet Stream), was run at the following ESI parameters: gas (N_2_) temperature 350°C, gas (N_2_) flow 8 l/min, nebulizer pressure 50 psi, sheath gas (N_2_) temperature 350°C, sheath gas (N_2_) flow 12 l/min and capillary voltage 3000 V. The MS was operated in the positive ion mode using Agilent MassHunter software. For the detection and quantification of m^1^A, time-segmented multiple reaction monitoring (MRM mode) was applied in order to ensure the separation of m^1^A from other methylated adenosine derivatives. The elution of m^1^A took place in the time segment from 5 to 8.5 min, while e.g. m^6^A could be detected in the last segment starting at 14 min, thus the segmentation allowed the exclusive detection of m^1^A. Mass transitions and QQQ parameters used can be found in Table 1. Peak areas were determined employing Agilent MassHunter Qualitative Analysis Software. In the case of adenosine, peak areas were extracted from the recorded UV chromatograms in order to avoid saturation of the mass signals.

**Table 1.** QQQ parameters of the dynamic MRM method

Mod.nucleoside	Precursorion [m/z]	Production [m/z]	Fragm.voltage [V]	Coll.energy [eV]	Cell accel.voltage [V]	Time segment [min]
^12^C-m^1^A	282	150	92	17	2	5–8.5
^13^C-m^1^A	293	156	92	17	2	5–8.5

##### m^1^A and A quantification

In order to quantify the m^1^A content of the RNA samples, ^13^C-labeled total RNA from *S. cerevisiae* was used as a stable isotope-labeled internal standard (SIL-IS) as described for total RNA from *Escherichia coli* previously (24). Briefly, 10 calibration solutions containing 0.01–500 fmol/μl m^1^A (Sigma Aldrich, Munich, Germany) and 10 ng/μl SIL-IS were prepared and analyzed by LC-MS/MS (injection volume 10 μl/sample). For determination of a nucleoside–isotope response factor for m^1^A, the ratio of the extracted areas of the ^12^C-m^1^A and ^13^C-m^1^A peaks was calculated for each calibration solution. The resulting response factor was then used for m^1^A quantification in the RNA samples.

Quantification of A was performed by running an external calibration series (5–1000 pmol) and extracting the peak areas from the recorded UV chromatogram. For inter-sample comparability, the detected m^1^A amount was normalized to the A content for each sample (% m^1^A/A). For the synthetic RNA samples with defined sequence, the quantification of A enables the calculation of the analyzed amount of RNA as well as the percentage of RNA molecules carrying an m^1^A modification. Results are displayed in Supplementary Table S7.

## RESULTS

Our approach to capture a comprehensive profile of reverse transcription encounters with m^1^A containing templates is depicted in Figure 1. Like in conventional RNA-Seq procedures, RNA preparations were reverse transcribed into cDNA libraries and submitted to Illumina sequencing. However, in contrast to typical library preparations, which include numerous biochemical steps prone to result in biased amplification of certain RNA species, we applied a specifically optimized protocol (18,20). This was designed to minimize such biases, as well as to capture abortive reverse transcription products originating in particular from encounters of the enzyme with nucleotide modifications in the template. The choice of RNA preparations as starting material was guided by the necessity to assess or eliminate, by proper control samples, other factors known to influence the RT-signature. Thus, we compared known m^1^A sites in tRNA and rRNA with that of null mutants to assess the influence of strongly structured RNA domains on the RT-profile. Short synthetic m^1^A containing oligonucleotides were included to assess the influence of the nucleotide directly 3′-to the m^1^A site, as this is the last one to be conventionally reverse transcribed before the direct encounter of the RT-enzyme active site with the m^1^A modification. For all known m^1^A sites, the resulting reads were inspected for their arrest rate at the m^1^A site, and in reads bypassing the modification site, the ratio of all four nucleotides was determined and extensively analyzed.

### Library preparation

An overview over the library preparation is given in Supplementary Figure S1. It was slightly adapted from a previously published protocol (18). The first step included in an optional fragmentation, applied to preparations containing RNAs significantly longer than tRNAs, such as e.g. rRNA. It consisted in incubation with ZnCl_2_, followed by excision from preparative PAGE of a size range denoted by the 50 and 150 nt bands of a size standard. Treatment with alkaline phosphatase was performed to remove phosphates and cyclic 2′-3′-phosphates that might block the 3′-end for the subsequent ligation with a DNA adapter. The adapter contained the following sequence elements (details in ‘Materials and Methods’ section): (i) pre-adenylated 5′-cytidine (18) (ii) nine randomized nucleotides (‘N’) for indexing of individual molecules (iii) P5 Illumina sequencing template (iv) 3′-blocking non-nucleosidic building block. In a third step, adapter ligation was followed by specific hybridization and elongation of a primer complementary to the non-random part of the adapter, resulting in a cDNA library. This library contained the pertinent information, namely the length and sequence of RT-events resulting from read-through events at the modification site, as well as of abortive products. To quantitatively convert this information into ds DNA libraries ready for Illumina sequencing, the RNA was degraded by alkaline hydrolysis, and the cDNA was submitted to CTP tailing by terminal transferase. The oligocytidine overhang was used as an anchor to hybridize a secondary adapter of double-stranded DNA, containing one helper strand in addition to the principle primer (18). The latter contained the following elements: (v) 5′-phosphate for ligation and (vi) the P7 Illumina sequencing element (sequences in Supplementary Table S1). The complementary helper strand contained an additional two guanosines as an overhang on its 3′-end to improve ligation efficiency by hybridization to the oligocytidine tail of the cDNA. This library was amplified in two PCR steps, the first one using only the P5 and P7 sequencing primers. After gel purification and excision, the second PCR was conducted using the full length P5 and P7 primers containing indices i5 and i7 for dual barcoding of multiple samples in a single sequencing run, as well as flow cell anchoring sequences. This latter step allows direct sequencing on the Illumina platform, circumventing an additional step, normally contained in the TruSeq kit protocol. A total of 20 libraries were prepared for this paper, annotated with various relevant characteristics as listed in Supplementary Table S2.

### Characterization of RT-signature at known m^1^A sites

To find particular m^1^A-related signatures in thus prepared RNA-seq libraries, the reads were mapped onto a minimal target genome consisting only of rRNA and tRNA sequences. The respective sequences have been obtained from the Modomics database (9) and do thus not contain any unspliced or unprocessed sequences, but, importantly, sequences of known modifications status. This is in some contrast to the previously published HAMR method (16), which relied on the generation of tRNA families form the ensemble of genetic copies of tRNA genes. The precise mapping parameters are of some concern, because among the various tRNAs sequences present in e.g. yeast, there are many strong similarities, especially among isoacceptors. Isoacceptors are tRNA species related by the amino acid they decode and are charged with on their 3′-end (25). As a results of such similarity, a significant fraction of reads may be assigned to targets that they do not biochemically originated from also outside the isoacceptor context. To evaluate the degree to which such mismapping might influence a potential m^1^A-signature, we used a parameter called the Levenshtein distance, essentially the number of mutation steps necessary to interconvert both species (26). As this is a measure of the relative similarity of two given RNA sequences, it inversely correlates with the probability for mismapping between the two species. The comparison of yeast tRNA sequences based on Levenshtein distance (details shown in Supplementary Figure S2) impressively shows, that most concerns for mismapping must be directed toward isoacceptors, while the sequence similarities outside these groups are minor in comparison. Therefore, for reads with multiple potential mapping sites, a regime termed ‘k1’ was applied, which reports one valid mapping site only. This and the treatment of other details on the mapping strategy must be relegated to the discussion part of this manuscript, because most of the relevant aspects are yet to be developed below. Thus, for example we later on comment on the clear advantages of the k1 regime when compared to the k3 regime (results displayed in Supplementary Figure S3), which reports up to three valid mapping sites.

**Figure 2.**
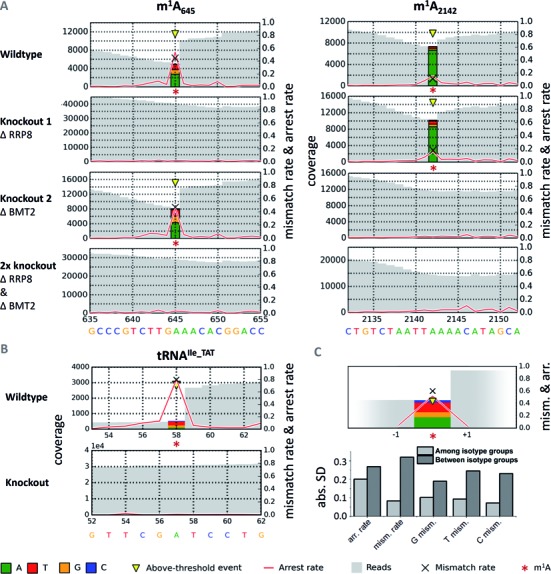
Detection of m^1^A signatures in deep sequencing data. The representations illustrate the coverage of a given site in gray, the arrest rate is plotted as a red line, and the mismatch composition is visualized by colored stacks at the m^1^A sites. For a position p, the arrest rate reflects the relative amount of mapped reads ending at p + 1, i.e. not covering p. (**A**) Sequencing profiles from single and double methyltransferase knockouts of *Saccharomyces cerevisiae*'s LSU rRNA with m^1^A sites 645 and 2142. Signatures of m^1^A residues are clearly apparent in the wild-type, and disappear in the corresponding knockout constructs. (**B**) Sequencing profiles of tRNA^Ile_TAT^ from wild-type and Trm6-knockout strains. The signature clearly disappears in RNA from a knockout strain of the enzyme, which is responsible for synthesis of m^1^A_58_ in tRNAs (28). tRNA^Ile_TAT^ was chosen as an example out of 37 signatures, which are detailed in Supplementary Figure S4. Positions are labeled according to absolute length of reference sequences, including variable regions. (**C**) Average signature of said 37 yeast cytosolic tRNAs at m^1^A_58_ complemented with absolute standard deviations of signature features among and between groups of isotypes. For the displayed profile, signatures were averaged among isotypes first, before calculating the final means.

To initially circumvent the above problems, a first assessment of RT-signatures was conducted with yeast 25S rRNA, which has known m^1^A sites at positions 645 and 2142 of the large ribosomal subunit (entry 5 in in Supplementary Table S2). Pure 25S rRNA, isolated from whole ribosomes as described in (6) showed a distinct occurrence of both, abortive RT-products and misincorporation of non-adenosine signals at positions suggestive of a causal connection to the presence of m^1^A. Importantly, both aspects were absent in negative controls obtained from either single or double knockout strains (6) of the methyltransferases responsible for the respective methylation (Figure 2A). Similarly, comparable RT-signatures were detected at position 58 of various yeast tRNAs, of which one example is shown in Figure 2B, whereas the remainder is detailed in Supplementary Figure S4 and an average signature is compiled in Figure 2C, which also lists the corresponding deviations. These signatures were absent in tRNA preparations from a knockout strain of the respective tRNA m^1^A methyltransferase (Figure 2B) (27,28). This clearly demonstrates that m^1^A residues leave a distinct signature even in RNA species whose stable structures are known to affect RT-arrest rates.

These RT-signatures displayed common characteristics in the mismatch incorporation of nucleotides into the cDNA at the positions corresponding to m^1^A in the RNA template. However, significant variation is evident, which also applies to the RT-arrest rate between m^1^A and the position to its 3′, as indicated by a red line in Figure 2. Clearly, a significantly larger number of instances must be investigated for a comprehensive picture. Therefore, we analyzed the RT-signatures of known m^1^A residues in further RNA preparations with known m^1^A sites, including yeast tRNA, human mitochondrial tRNA, human rRNA and rRNA from *S. pactum* (samples listed in Supplementary Table S2). The latter is of particular interest, because its m^1^A residue, which mediates an antibiotic resistance, is the only one situated in small subunit rRNA.

### m^1^A's RT signature is dependent on the sequence context of the RNA template

**Figure 3.**
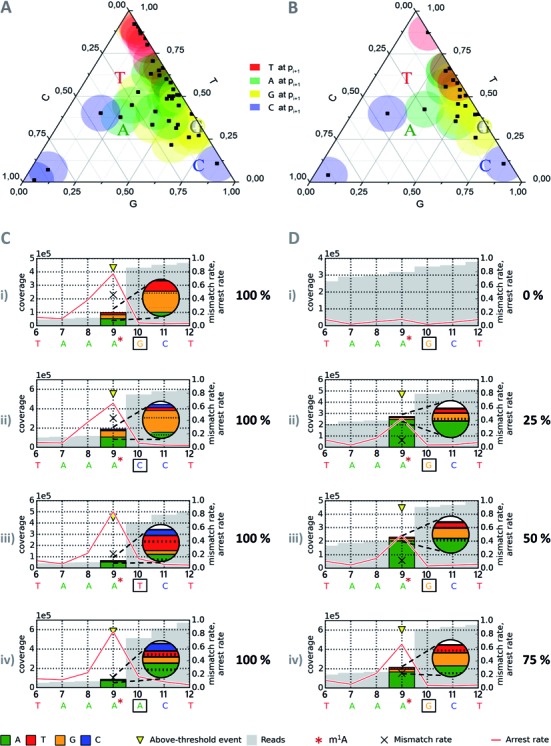
Revolver assay. Revolver oligonucleotides feature permutation of the four major nucleotides at a position of interest, here the +1 position (3′ to m^1^A). For a position p, the arrest rate reflects the relative amount of reads ending at p + 1 (i.e. not covering p) out of all reads covering p + 1. (**A**) Ternary plot of mismatch composition of 41 natural m^1^A sites (black dots) for base configurations guanosine (yellow), cytidine (blue), uridine (red, T in mapping profile) and adenosine (green) at position +1 w.r.t. m^1^A. Data points from revolver oligonucleotides are represented as colored letters corresponding to the color code also used in (**C**) and (**D**). (**B**) Twenty-two hierarchically clustered data points derived from initial 41 measurements in (A). (C) Mismatch composition at m^1^A site for base configurations guanosine (i), cytidine (ii), uridine (iii, T in mapping profile) and adenosine (iv) at position +1 w.r.t. m^1^A in sequencing profiles of synthetic oligonucleotides. (D) RT signature by modification level. Arrest rates and mismatch contents at different ratios of modified and unmodified equivalents of revolver oligonucleotide are shown: 0% m^1^A in (D-i), 25% in (D-ii), 50% in (D-iii), 75% in (D-iv) and 100% m^1^A in (C-i).

For all instances from Table 2, the m^1^A-dependent mismatch composition was analyzed as a function of the neighboring sequence context, including one upstream nucleotide and two downstream nucleotides of the RNA template, denoted −1, +1 and +2, respectively. This implies that nucleotides +1 and +2 are reverse transcribed, before the m^1^A residues acts as a template in the RT active site, with +1 denoting the characteristic position after which RT-arrest occurs. Consequentially, the −1 position only enters the RT active site after the enzyme has bypassed the m^1^A residue. In a first instance, the influence of each position was analyzed independently from the others. A distinct influence of a given nucleotide would result in a clustering of signals in a ternary plot (16) of the mismatch composition. While no significant impact of nucleotide identity on positions −1 and +2 was observable in such plots (Supplementary Figure S5 A and C), the ternary plot of position +1, visualized in Figure 3A and B, stands out. For example, the 5′-m^1^A-U-3′ motif leads to very efficient misincorporation of dATP into cDNA.

**Table 2.** m^1^A sites

RNA spec.	Position	Organism	Distinct RNAs	Replicates
**Confirmed**				
tRNA cyt.	58	Yeast	20	2
tRNA mit.	9	Human	13	1
rRNA	645	Yeast	1	2
rRNA	2142	Yeast	1	2
rRNA	1309	Human	1	1
rRNA	964	*Streptomyces pactum*	1	1
Artif. oligo.	9*		2	2
Revolver oligo.	9*		4	1
tRNA^Arg_UCG^ cyt.	58	*Trypanosoma brucei*	1	2
**Unconfirmed**				
rRNA	1136	Mouse	1	2
tRNA mit.	9	Human	1	1
tRNA cyt.	58	*Trypanosoma brucei*	15	1

Confirmed instances include published and self-designed m1A sites, whereas unconfirmed sites rely on homologous identification. Distinct RNAs refers to the number of non-redundant RNAs, in which m1A signatures were found. * labeled synthetic oligoribonucleotides contain m1A9 in a sequence derived from tRNALys of *Homo sapiens*.

Since nucleotide information is mapped to the template sequence, this corresponds to high T signal, as well as to low G and low C signals. This characteristic misincorporation pattern is visually recognizable by clustering of 5′-m^1^A-U-3′ derived red data points in the upper end of the ternary diagram in Figure 3A. Similarly, m^1^A-G (yellow) and m^1^A-A (green) give rise to distinct clusters with overall low C signal, while data points for m^1^A-C are more spread out, and share high cytidine and low thymidine content as a common characteristic.

Of the originally 54 m^1^A instances present in our data, experimental replicates were averaged, leading to the 41 data points plotted in Figure 3A. Because certain sequence contexts were over-represented in that dataset, we further reduced the dataset by averaging data points from RNAs of over 95% sequence identity (e.g. tRNA sequences containing SNPs) and a final averaging step left only sequences differing at positions −1, +1 and +2, relative to the m^1^A site. This dataset, which is plotted in Figure 3B, only incompletely covers the permutation space of said three positions (Supplementary Figure S5 D). Therefore, to cover more of the remaining sequence space, we investigated the RT profiles of synthetic m^1^A-containing oligoribonucleotides. These oligoribonucleotides were derived from the naturally occurring m^1^A containing sequence of human mitochondrial tRNA^Lys^ (11). In what we termed ‘revolver’ concept, position +1 was systematically variegated, such that the influence of the respective nucleotide could be assessed in direct comparison. The resulting RT-profiles, which are visualized in Figure 3C, point out a pronounced effect of position +1. As is apparent by visual inspection of Figure 3A and B, in which the revolver data are highlighted by colored letters, they reflect well the clustering of the respective natural instances. This visually apparent clustering was statistically verified (computational details in Supplementary Figure S5 E, F and Method S1). Further computational inspection of the revolver-extended experimental dataset (as detailed in the supplement) did not reveal any significant influence of positions +2 and −1. Note that all revolver oligonucleotides in Figure 3B show similar arrest rates, suggesting that the sequence context at the +1 position does not significantly influence the reverse transcription arrest rate.

### Quantification of m^1^A occupancy

Synthetic oligoribonucleotides were also used to gauge the effect of incomplete occupancy of the modification site. Figure 3D shows profiles obtained from the unmodified oligoribonucleotide of wild-type sequence mixed with increasing amounts of the corresponding m^1^A containing oligoribonucleotides. Clearly visible, both, the arrest rate and the misincorporation increase linearly with the fraction of m^1^A. This suggests, that some of the biological samples might be incompletely modified and that RT-profiles may eventually be used to gauge modification efficiency after thorough calibration. Therefore, in addition to verifying the presence or absence of m^1^A, we have quantified the m^1^A content by LC-MS, using a recently developed biosynthetic stable isotope labeled standard (24).

**Figure 4.**
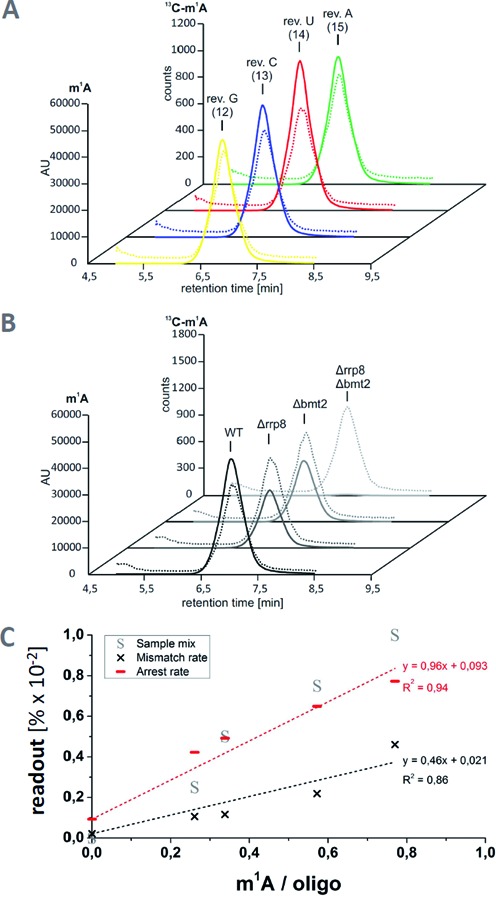
Quantification of m^1^A by LC-MS using a biosynthetic internal standard. LC-MS/MS chromatograms showing the m^1^A and ^13^C-labeled m^1^A peaks in the revolver oligonucleotides (**A**) and in 25S rRNA from wild-type and rrp8/bmt2 knockout yeast (**B**). Continuous lines represent the peaks of unlabeled m^1^A, dotted lines those of ^13^C-labeled m^1^A added as an internal standard (24). To ensure inter-sample comparability of the m^1^A peaks, the peak heights were adjusted to the respective ^13^C-m^1^A peaks and normalized to the injected amount of oligonucleotide or 25S rRNA. The amount of analyzed oligonucleotide or 25S rRNA was determined by calculating the amount of adenosine in the respective samples using the UV peak of adenosine and dividing the amount by the number of adenosines per molecule. AU—arbitrary units. (**C**) Plot of RT signature occupancy by m^1^A content.

Figure 4 shows chromatograms of the four revolver-oligoribonucleotides, from which an m^1^A content of about 80± 10% at position 9 was calculated. Only traces of m^6^A, a known rearrangement product of m^1^A, were found, therefore a possibility of incomplete occupancy at m^1^A_9_ even in synthetic samples remains. Not surprisingly, a plot of mismatch rate and arrest rate as a function of m^1^A content in Figure 4C suggests a linear dependence of both parameters, but neither correlation is precise enough to confirm or discard the possibility of incomplete m^1^A modification in the revolver oligoribonucleotides. LC-MS quantification of the m^1^A sites (Figure 4B) in yeast rRNA by analysis of the single knockouts and double knockout yielded 0.7 mol m^1^A per mol rRNA for each of both sites, which is consistent with a total of 1.4 mol m^1^A per mol rRNA in the wild-type. Interestingly, the profiles vary strongly, although both sites have similar occupancy. Thus, while arrest and mismatch rate at position m^1^A_645_ (Figure 2) correlate at least roughly with the m^1^A content, the profile at m^1^A_2142_ incorrectly suggests a much lower modification occupancy. Of note, LC-MS analysis (Figure 4B) and RNA-Seq analysis were conducted with aliquots from the same rRNA preparation, and while the quantification of fractional occupancy is apparently fraught with a ∼10% error in precision, the relative comparison of rRNA from both knockout mutants is largely more precise, because numerous error sources average out (24). Comparison of the profiles of m^1^A_645_ and m^1^A_2142_ clearly show differences despite identical occupancy. In the case of m^1^A_2142_, a large fraction of correctly incorporated dTTP, visualized as green bar, erroneously suggests a significant fraction of unmodified A residues in the RNA template. This leads to the conclusion that RT-profiles have limited use in the quantification of fractional occupancy, in that they tend to underestimate the degree of fractional occupation because the RT does occasionally incorporate the correct dTTP even when challenged by m^1^A. Inversely however, the sum of RT-arrest and misincorporation provides a plausible lower limit, since these events clearly derive from events occurring only on an m^1^A containing RNA template. This finding has deeper implications and consequences, in particular for structural probing experiments with DMS.

### Confirmation of m^1^A sites predicted by homology

**Figure 5.**
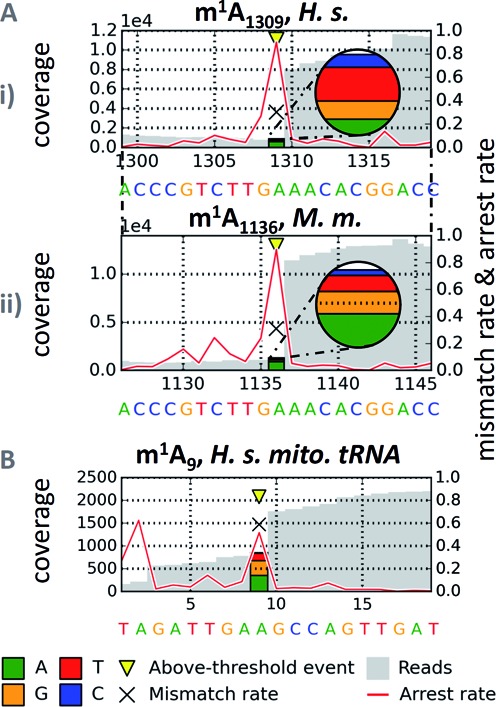
Homology based confirmation of m^1^A. For a position p, the arrest rate reflects the relative amount of mapped reads ending at p + 1, i.e. not covering p. (**A**) Homologous identification of m^1^A_1136_ in murine 28S rRNA (i) by alignment to human sequence containing m^1^A_1309_ (ii). (**B**) m^1^A_9_ in human mitochondrial tRNA, identified by alignment to identical bovine sequence with published m^1^A_9_.

Despite the variations described above, the signature of m^1^A appears characteristic already by visual inspection of RNA-Seq representations in Figures 2 and 3. Of obvious interest is the use of such data for the detection of m^1^A residues where they have not been detected by other methods. Arguably the easiest application is the qualitative confirmation of m^1^A at putative sites that show plausible homology to known sites. For example, human 28S rRNA was reported to contain an m^1^A residue at position 1309 (29), while the corresponding rRNA from mouse has not yet been analyzed for this modification. Figure 5 shows a strong m^1^A signature at the corresponding position in human rRNA, as well as at position 1136 of mouse rRNA, which is homologous to the human site. Another example is the m^1^A signature at position 9 of human mitochondrial tRNA^Asn^ (Figure 5B), of which the bovine homolog has recently been sequenced (30). These profiles plausibly show that an RT-signature can qualitatively confirm the presence of m^1^A.

To apply this identification by computer-aided visual inspection in a more challenging biological question, we have applied it to an organism in which the occurrence of m^1^A in tRNA was little investigated, namely *T. brucei*. From a dataset obtained by application of the library preparation protocol outlined above to total RNA (22), we isolated the profiles of tRNAs. By visual inspection, 16 species showed a clear m^1^A signature, as shown in Supplementary Figure S10A. Importantly, the sequence dependence of their mismatch distribution, which is plotted in Supplementary Figure S10C, agrees very well with the authentic one in Figure 3B. To further verify the actual existence of m^1^A in at least one of these species, we isolated tRNA^ARG_UCG^ by hybridization with a biotinylated cDNA, and subsequent sequestration on streptavidin-beads, as detailed in the ‘Materials and Methods’ section. The purified tRNA was submitted to both-LC-MS analysis and renewed RNA Seq. Both confirmed the presence of m^1^A. LC-MS analysis suggested near complete occupancy, i.e. one m^1^A residue per tRNA molecule (Supplementary Table S7), and the m^1^A signature obtained from the isolated tRNA (Supplementary Figure S10B) is in excellent agreement with that of the bulk tRNA, experimentally confirming that mismapping effects are indeed minor.

### Supervised prediction of m^1^A by machine learning

To quantitatively assess how robustly m^1^A signatures can distinguish actual modification sites from non-m^1^A sites in RNA-Seq data, a supervised prediction of m^1^A by machine learning was conducted. Known instances of m^1^A and non-m^1^A sites in equal numbers were fed to a machine learning algorithm (overall workflow depicted in Supplementary Figure S6). Thus, 45 m^1^A signatures (coverage ≥ 10, 3′-adjacent coverage ≥ 15, taking isoacceptors into account as separate entities) of tRNA, rRNA and artificial oligonucleotides were merged with an equal amount of data points from non-m^1^A sites randomly drawn from the adenosine pool of *bona fide* m^1^A-containing datasets: mitochondrial (human) and cytosolic (yeast) tRNA and rRNA (yeast and mouse), according to setting (i) in the ‘Materials and Methods’ section. This dataset was fed to a RF model (500 trees) classifying both kinds of adenosine instances. Briefly, an RF (31) is a machine learning model for object classification by an ensemble of decision trees. Under randomization in training, binary forks are formed in each individual tree, used to differentiate objects according to their features, based on information content. The final object classification is a consensus of all class votes returned by the single trees. Features visible to the RF-classifier included: arrest rate *a*, mismatch rate *m*, the *m*/*a* ratio, the mismatch composition (fractions of G, T and C), and a parameter that we termed CSA. The latter is defined as the fold change of the site's *a* with respect to its sequence environment of five bases up- and five bases downstream (details in the ‘Materials and Methods’ section).

**Figure 6.**
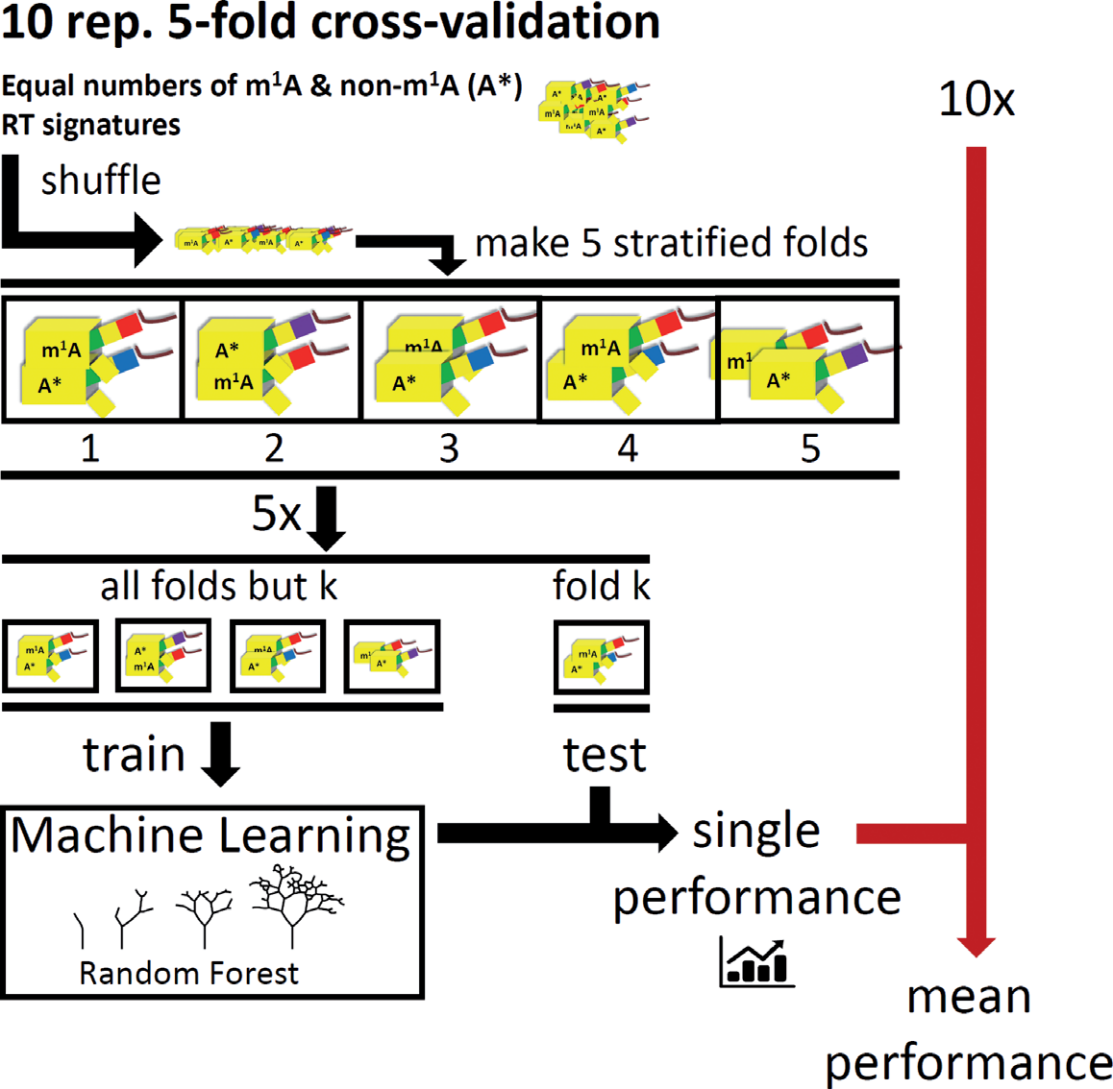
Validation outline for supervised prediction. RT signatures (yellow) of m^1^A and non-m^1^A (A*) sites and are distributed into subsamples, termed folds, with uniform ratios (stratification) m^1^A / A*. The system was tuned toward both, sensitivity and specificity by equal abundance of each class, minimizing learning biases due to *a priori* class probabilities. In each of 10 repetitions (10×), the Random Forest was trained on another four of five possible fold combinations (5×) and tested on the respective left-out fold.

Despite its documented impact we did not include the identity of the +1 neighboring base, in order to avoid biases and overfitting side effects in the training run. We applied a five-fold stratified cross-validation (Figure 6), i.e. the data were divided into parts parts, of which four-fifth were used for a training run and one-fifth as a test dataset, for which the model was tasked to classify all adenosines. In a total of 10 runs, during which the five parts were permutated between training and testing sets, the model scored better than 97% for both, sensitivity and specificity (setting (i), detailed in ‘Materials and Methods’ section). In setting (ii), a more stringent variation of this validation, the non-m^1^A sites fed to the RF were deliberately chosen among those that showed the closest resemblance to m^1^A-signatures among the non-m^1^A sites. Under these circumstances, the values dropped to 89% (SD = ±2.4%) for sensitivity, and 87% (SD = ±2.8%) for specificity (averaged from ten repetitions, all statistics are given in Supplementary Table S5). Of interest is the deliberate inclusion of other modified adenosine residues in the training set for ‘non-m^1^A’, in particular of two ubiquitous consecutive m^6,6^A rRNA residues at positions 1781 and 1782 of yeast 18S rRNA. This modification type features a methyl group on the Watson–Crick face, as does m^1^A and was reported to show a similar misincorporation pattern (16). Indeed, m^6,6^A_1781_ shows a signature (shown in Supplementary Figure S9) that, by visual inspection, is indistinguishable from that of m^1^A (Figure 2C) and is classified as such by the algorithm as well. On the other hand, the adjacent m^6,6^A_1782_ also shows a clear signature, which is however, different from the typical m^1^A signature. Accordingly, it is correctly classified as ‘non-m^1^A’ by the algorithm. Of note, when the model was trained on the entire amount of available tRNA instances of m^1^A with as many random non-m^1^A adenosine signatures (setting (iii)), all presented (five) rRNA sites of m^1^A (2× *S. cerevisiae*, 1× *S. pactum*, 1× *M. musculus*, 1× *H. sapiens*) were correctly identified with a specificity >99.9%.

In addition, a leave-one-out cross-validation was performed. This roughly corresponds to the above stratification concept with the number of folds maximally increased, such that the test-fold contains only a single positive and a single negative m^1^A instance. As expected, performance increased with availability of additional training instances (Supplementary Table S6). This thus provides additional confirmation of the overall feasibility of the concept and underlines the need to maximize training instances for efficient machine learning.

Out of concern that our dataset of 45 positive m^1^A instances might be too limited for complex classifiers such as RF, we compared the performance of the latter with a more basic method, namely k-Nearest Neighbor (kNN). In a so called receiver operating characteristic (ROC) analysis, the area under the curve corresponds to the probability with which a model scores a random positive m^1^A instance higher than a negative one. As can be deduced from the various curves plotted in Supplementary Figure S7, the RF model consistently outperforms various kNN setups by a large margin. The shape of the RF curve also illustrates that the RF achieved considerable sensitivity while maintaining high specificity.

Finally, we have analyzed in depth, which parameters have been retained by the RF model as most informative for the recognition of m^1^A sites. From inspection of the trained RF, it became clear, that both, arrest rate and at mismatch rate played an important role for performance. A more detailed analysis was conducted by a leave-feature-out analysis, which measures performance in various permutations of incomplete feature combinations. The results, which are shown in Supplementary Figure S8, clearly confirm our initial approach to m^1^A signature identification, namely that neither arrest rate nor mismatch analysis alone come close to the performance of their combination.

## DISCUSSION

Encounters of reverse transcriptase enzymes with non-canonical nucleotides are about to become a focus of intense research. Early investigations into the effect of m^1^A were mostly concerned with the application in structural probing *in vitro* (1), and the replication of the HIV genome *in vivo*, which actually strongly relies on RT-arrest induced by m^1^A_58_ of the HIV-primer tRNA^Lys3^ (32–34). The topic is subject to renewed impetus, as RNA-Seq based approaches are being developed to detect RNA modifications on a transcriptome wide scale. Such analyses for m^5^C (35), m^6^A (36) and pseudouridine (37–39) have recently revolutionized the RNA modification field, and the common belief is that more modifications types are to be found in transcriptomes by similar methods. So-called PSI-Seq (37–39) relies completely on RT-arrest upon encounter of the enzyme with a CMC modified nucleoside and an understanding of the efficiency of RT-arrest clearly will improve the accuracy of such approaches.

Here, we present an in-depth investigation of the effect of m^1^A in an RNA template on the composition of cDNA fragments generated during reverse transcription. Where previous studies have provided a more general picture of various modifications in parallel (16,17), we focused on a single modification species and characterized the resulting arrest rate as well as the misincorporation pattern for over 50 RNA sequences. We taught the common characteristics to a computer learning program for supervised prediction and identification. The method is well capable of qualitatively confirming the presence of m^1^A at a defined candidate position, such as e.g. A_964_ in rRNA from *S. pactum*. Since this methylation mediates resistance to the pactamycin (12), our approach can conceivably be applied to the detection of antibiotic resistance. Because m^6,6^A blocks the Watson–Crick face of an adenosine like m^1^A and leaves signatures as well, we expect that moderate adaptation of parameters will allow the monitoring of m^6,6^A at position 1519 in bacterial rRNA, which mediates resistance to kasugamycin (40). From our results, we can project that the limiting step in this endeavor is likely to be a larger training set of *bona fide* m^6,6^A sites.

### Parameters that shape the RT-signature

As an important message, the presented data suggest, that the amount of misincorporation by the RT enzyme is very substantial, resulting in a non-negligible read-through efficiency. It is known from the literature, that read-through by RT-enzymes *in vitro* may depend on a variety of parameters, including e.g. the dNTP concentration (41) in the case of 2′-OMe modifications (42). Certainly, the nature of the enzyme itself is important in the encounter with an RNA modification (43), and we can expect key parameters of *in vitro* conditions such as pH, ion strength and divalent cations to be important as well. The present study has kept these parameters constant and focused on the identification of features residing in the RNA template itself. Our investigations into the influence of neighboring nucleotides −1, +1 and +2 revealed a clear influence of the nature of the +1 nucleotide, situated 3′ to the m^1^A residue. Beyond the scope of detecting m^1^A residues at new positions in transcriptomes, this insight has significant implications for the interpretations of structural probing data obtained by primer extension. With respect to the interpretation of structural probing data of m^1^A residues generated using DMS, the classification of RT-arrest signals as weak, intermediate, or strong (44), may now be refined by taking into account the penultimate nucleotide.

Higher order structure of RNA has long been known to negatively affect the efficiency of primer extension, a fact frequently encountered in structural probing of e.g. rRNA, where a strong noise from RT-arrest signals made data interpretation difficult. Our data, however, suggest that RNA structure may, in certain cases, even facilitate read-through. This is exemplified by comparison of the signatures of rRNA (Figure 2) with revolver oligonucleotides (Figure 3). The latter can reasonably be assumed to be weakly structured (11). In each case, the m^1^A residue causes >80% arrest rate, while the two rRNA sites show strongly diverging arrest albeit being equally modified to ∼70%. Said discrepancy must mostly stem from outside the immediate neighboring sequence context, which is quite similar between both sites.

### Limitations

From the above discussion flow a number of limitations of the presented method at its current state. Clearly, estimates of fractional occupancy of an m^1^A site can be semi-quantitative at best, and only after calibration as shown in Figure 3D. The differential strength of equally modified sites in rRNA (Figure 2A) points to further factors that influence the strength of the m^1^A signature, whose identification must await further work. In contrast, analyses of different mapping strategies as detailed in Supplementary Figure S3 show, that the influence of the mapping strategy was efficiently minimized in the k1 regime we applied. This analysis revealed that for tRNA-related reads, mismapping in a k3 regime, which allows up to three mappings per read, strongly depends on the tRNA species, and may potentially outnumber the reads derived from conservative k1 regime mapping by more than an order of magnitude (Supplementary Figure S3A), although the k3-related mismapping was mostly inside isoacceptor groups. Interestingly, differences in the signature-relevant parameters arrest rate and mismatch content were relatively minor (Supplementary Figure S3C), when k1, k3 and ‘best’ (default) settings were compared, and this was exemplarily visualized for a selected tRNA species in Supplementary Figure S3D. For scenarios with higher cross-mapping rates, reporting the best alignment on cost of computation time may be considered, although this can lead to undesired suppression of mismatch information. In this study, variation of signature parameters due to mapping artefacts is clearly smaller than what we expect from experimental parameters. Given that salt conditions, temperature and the type of enzyme are known to affect polymerization characteristics e.g. in PCR reactions (45), we will turn our attention to these parameters in the near future.

### Potential applications, prediction performance and scope

In the rapidly developing field of RNA modifications there is an urgent need for new methods, which are sought for applications to a variety of biological questions. These include transcriptome-wide searches to detect new modifications sites as well as quantification e.g. in the context of a response to outside stress. With respect to the latter, elevated temperatures were recently shown to ablate a thiol-modification in yeast (46,47). In analogy, we have investigated potential changes in the m^1^A-signatues of tRNAs from yeast raised at normal temperature versus 39°C, but failed to identify any differences (data not shown). Although this might be due to the limited quantification accuracy (compare Figure 3D and related material), total ablation would have been detectable.

At the current state, our machine learning algorithm can distinguish m^1^A from an unmodified adenosine with very good accuracy, if these two possibilities are the only elements in the training data. Not unexpectedly, when other modifications are forcibly included as non-m^1^A training data, the performance drops. The erroneous classification of an m^6,6^A as m^1^A is readily rationalized: m^6,6^A carries a methyl group on its Watson–Crick face, and therefore leads to RT-arrest as well. Furthermore, a previous study suggests that all the adenosine modifications have similar misincorporation patterns (16). The latter argument must be attenuated somewhat, since our current analysis shows strong variability even within m^1^A samples (Supplementary Figure S4). Still, this instance once more illustrates, that once a candidate site is identified, further evidence, such as sequence homology to known sites, RNA-Seq data from relevant knockout organisms, or biochemical analysis is needed for confirmation.

The performance of a prospective large-scale prediction depends on the quality and quantity of both, positive and negative training instances. Our m^1^A pool covers a large number of sequence contexts, but is clearly biased in that some portions of the sequence space are missing in the training pool. Obviously, the sequence context of m^1^A occurrence in nature is not random, but biased by biological evolution, e.g. of the m^1^A methyltransferases (48,49). Since the algorithm is based on learning, its current version will be more successful at predicting m^1^A sites situated in a similar sequence context, and it is prone to perform poorly in the prediction of sites in a radically new sequence context, including in particular such situated in clusters containing multiple different modifications. The training pool of non-m^1^A instances determines the success along similar lines.

## Supplementary Material

SUPPLEMENTARY DATA
