# Supplementary material for: The reverse transcription signature of N-1-methyladenosine in RNA-Seq is sequence dependent
Source: Nucleic Acids Res. 2015 Sep 13;43(20):9950–64. doi: 10.1093/nar/gkv895 (PMC4787781; doi:10.1093/nar/gkv895)
Supplement: SUPPLEMENTARY DATA [file supp_43_20_9950__index.html]

The reverse transcription signature of N-1-methyladenosine in RNA-Seq is sequence dependent — The reverse transcription signature of N-1-methyladenosine in RNA-Seq is sequence dependent — SUPPLEMENTARY DATA 

# The reverse transcription signature of *N*-1-methyladenosine in RNA-Seq is sequence dependent

## SUPPLEMENTARY DATA

- SUPPLEMENTARY DATA
