# Supplementary material for: The reverse transcription signature of N-1-methyladenosine in RNA-Seq is sequence dependent
Source: Nucleic Acids Res. 2015 Sep 13;43(20):9950–64. doi: 10.1093/nar/gkv895 (PMC4787781; doi:10.1093/nar/gkv895)
Supplement: SUPPLEMENTARY DATA [file supp_gkv895_nar-01052-z-2015-File008.doc]

**Supplementary material to**

**The reverse transcription signature of *N*-1-methyladenosine in RNA-Seq is sequence dependent**

Ralf Hauenschild^1^, Lyudmil Tserovski^1^, Katharina Schmid^1^, Kathrin Thuering^1^, Marie-Luise Winz^2^ , Sunny Sharma^3^, Karl-Dieter Entian^3^, Ludivine Wacheul^4^,  Denis L.J. Lafontaine^4^, James Anderson^5^, Juan Alfonzo^6^, Andreas Hildebrandt^7^, Andres Jäschke^8^, Yuri Motorin^9^,* and Mark Helm^1^,*

**Table of Contents**

Page

**Library preparation** - Sequence elements **Tab. S1** 2

**Library preparation** – Outline **Fig. S1** 3

**Sequence libraries** **Tab. S2** 4

**Pairwise Levenshtein edit distances of cytosolic tRNA sequences** of *S. cerevisiae* **Fig. S2** 5

**RT signature vs. multiple mappings** **Fig. S3** 6

**m^1^A_58_ signature compilation** **Fig. S4** 7

**RT sequence context** **Fig. S5** 8

**Computational analysis of sequence context mediated mismatch composition** **Met. S1** 9

**Workflow** - Data processing for prediction **Fig. S6** 10

**Profile format** **Tab. S3** 10

**Classification input format** **Tab. S4** 10

**Random Forest performance** - 10 rep. 5-fold cross-validation **Tab. S5** 10

**Random Forest performance** - 10 rep. leave-one-m^1^A-out cross-validation **Tab. S6** 10

**Receiver operating characteristic** (ROC) **Fig. S7** 11

**Prediction quality vs. number and category of predictors** **Fig. S8** 12

**RT signatures of m^6,6^As 1781 and 1782** **Fig. S9** 13

**Trypanosomal m^1^A_58_** **Fig. S10** 14

**LC-MS/MS quantification** of m^1^A **Tab. S7** 15

**Table S1: Library preparation - Sequence elements.**

| **Element** | **Sequence** | | | | |
| --- | --- | --- | --- | --- | --- |
| RAdapter | 5’-P-CNNNNNNNNNAGATCGGAAGAGCGTCGTGTAGGGAAAGAGTGT-3‘-C6-spacer | | | | |
| RTPrimer | 5’-ACACTCTTTCCCTACACGACGCTCTTCCGATCT-3’ | | | | |
| DAnchorA | 5’-GTGACTGGAGTTCAGACGTGTGCTCTTCCGATCTGG-3’ | | | | |
| DAnchorB | 5’-P- AGATCGGAAGAGCACACGTCTGAACTCCAGTCAC-3’-C6-spacer | | | | |
| PCR P7 primer | 5’-CAAGCAGAAGACGGCATACGAGAT77777777GTGACTGGAGTTCAGACGTGTGCTCTTCCGATCT-3’ | | | | |
| PCR P5 primer | 5’-AATGATACGGCGACCACCGAGATCTACAC55555555ACACTCTTTCCCTACACGACGCTCTTCCGATCT-3’ | | | | |
| **Barcodes used with P7** | | **Sequence** | | **Barcodes used with P7** | **Sequence** |
| N701 | | TCGCCTTA | | N501 | TAGATCGC |
| N702 | | CTAGTACG | | N502 | CTCTCTAT |
| N703 | | TTCTGCCT | | N503 | TATCCTCT |
| N704 | | GCTCAGGA | | N504 | AGAGTAGA |
| N705 | | AGGAGTCC | | N505 | GTAAGGAG |
| N706 | | CATGCCTA | | N506 | ACTGCATA |
| N707 | | GTAGAGAG | | N507 | AAGGAGTA |
| N708 | | CCTCTCTG | | N508 | CTAAGCCT |
| N709 | | AGCGTAGC | |  |  |
| N710 | | CAGCCTCG | |  |  |
| N711 | | TGCCTCTT | |  |  |
| N712 | | TCCTCTAC | |  |  |
| **Oligoribonucleotide type** | | | **Sample ID** | **Sequence** | |
| A-G | | | 17 | 5’-CACUGUAAAGCUAACUUAGC-3’ | |
| revolver m^1^A-G | | | 12 | 5’-CACUGUAAm^1^AGCUAACUUAGC-3’ | |
| revolver m^1^A-C | | | 13 | 5’-CACUGUAAm^1^ACCUAACUUAGC-3’ | |
| revolver m^1^A-U | | | 14 | 5’-CACUGUAAm^1^AUCUAACUUAGC-3’ | |
| revolver m^1^A-A | | | 15 | 5’-CACUGUAAm^1^AACUAACUUAGC-3’ | |
| hybridization oligo for tRNA^Arg_UCG^ | | | S24 | biotin-CGGCAGGACTCGAACCTGCAACCCTCA | |

**Figure S1: Library preparation - Outline.** Note that the presented procedure captures cDNA resulting from abortive reverse transcription events. Primer information is given in Table S1.

**Table S2:** **Sequence libraries.** *Raw reads* denote the number of FASTQ sequences obtained from Illumina prior to bioinformatic processing. *Mapped* reads reflect the relative number of processed reads mappable to references provided to Bowtie2. Value pairs refer to reads from paired end libraries or replicates (sample 1). *Bp* is the length of these reads in base pairs, where 150 *bp* were used in the single end (s) mode of sequencing and 151 or 35 + 88 *bp* for the reads in paired end (p) mode of libraries. For comparability, the average number (*Ø reads / kb ref*) of reads mapped on a kilobase (*kb*) of an m^1^A-annotated target reference sequence is listed, e.g. tRNA^Ini^ in sample 3 and rRNA in sample 5. The mean coverage (*Ø cov. 3’ of m^1^A*) at +1 positions 3’ of m^1^A provides a reference for the arrest rate, (*Ø arr. (m^1^A)*), at the S*m^1^A* position and *Ø mism. (m^1^A)* provides the mismatch contents. Sample 1 values originate from replicates (N=2). Numbers annotated with ^Ini^ refer to tRNA^Ini^ only. Entries in brackets refer to pairs of m^1^A sites in the corresponding sample, such as m^1^A_645_ and m^1^A_2142_ in rRNA. The mean is given in front of each bracket.

| **ID** | **Interest** | **Material** | **Organism** | **Raw reads** | **Mapped [%]** | **Bp** | **End** | **Ø Reads / kb ref** | **Ø *cov.* 3’ of m^1^A** | **Ø *arr.* (m^1^A) [%]** | **Ø *mism.* (m^1^A) [%]** |
| --- | --- | --- | --- | --- | --- | --- | --- | --- | --- | --- | --- |
| 1 | Signature pool | total tRNA | *S. cerevisiae* | 2.95 M / 2.23 M | 40.0 / 40.7 | 151 | p | 370 k / 260 k | 10.0 k / 6.6 k | 21 / 22 | 54 / 54 |
| 2 | Signature pool | total mito. RNA | *H. sapiens* | 2.1 M | 14.3 | 151 | p | 189 k | 1.6 k | 62 | 32 |
| 3 | m^1^A_58_ knockout | total tRNA | *S. cerevisiae* Δ trm6 | 5.58 M | 89.9 / 89.1 | 35, 88 | p | 16.4 M^Ini^ | 776 k^Ini^ | 0.1^Ini^ | 0.4^Ini^ |
| 4 | Positive control | total tRNA | *S. cerevisiae* | 6.37 M | 77.3 / 82.1 | 35, 88 | p | 579 k^Ini^ | 1.25 k^Ini^ | 18.1^Ini^ | 7.0^Ini^ |
| 5 | Positive control | rRNA | *S. cerevisiae* | 4.95 M | 47.9 | 150 | s | 213 k | 9.3 k (10.0 / 8.6 k) | 35 (54.0 / 15.0) | 28.0 (44.7 / 11.2) |
| 6 | single knockout m^1^A_645_ | rRNA | *S. cerevisiae* Δrrp8 | 5.40 M | 63.1 | 150 | s | 407 k | 25.6 k (39.1 k / 12.0 k) | 9.15 (2.0 / 16.3) | 9.6 (1.2 / 18.0) |
| 7 | single knockout m^1^A_2142_ | rRNA | *S. cerevisiae* Δbmt2 | 3.82 M | 76.4 | 150 | s | 308 k | 12.6 (13.7 k / 11.6 k) | 23.35 (44.7 / 2.0) | 24.3 (47.0 / 1.6) |
| 8 | double knockout m1A_645_ and m1A_2142_ | rRNA | *S. cerevisiae* Δrrp8 + Δbmt2 | 7.37 M | 68.33 | 150 | s | 402 k | 21.6 k (28.3 / 14.8 k) | 2.75 (3.2 / 2.3) | 0.8 (0.8 / 0.8) |
| 9 | m^1^A on SSU of rRNA | total RNA | *S. pactum* | 6.27 M | 1.2 / 0.6 | 35, 88 | p | 250 k | 6.4 k | 76.0 | 56.9 |
| 10 | Homologous identification | rRNA | *H. sapiens* | 5.14 M | 77.7 / 72.0 | 35, 88 | p | 12 k | 8.0 k | 90.0 | 30.3 |
| 11 | Homologous identification | rRNA | *M. musculus* | 7.18 M | 71.0 / 68.7 | 35, 88 | p | 150 k | 11.4 k | 89.7 | 30.9 |
| 12 | RT sequence context dependency | oligo. | synthetic | 1.42 M | 92.6 / 86.8 | 35, 88 | p | 18.6 M | 430 k | 76.0 | 48.9 |
| 13 | RT sequence context dependency | oligo. | synthetic | 1.66 M | 87.9 / 82.3 | 35, 88 | p | 23.9 M | 550 k | 54.4 | 56.7 |
| 14 | RT sequence context dependency | oligo. | synthetic | 1.56 M | 84.1 / 80.1 | 35, 88 | p | 20.1 M | 410 k | 82.7 | 24.5 |
| 15 | RT sequence context dependency | oligo. | synthetic | 1.89 M | 88.4 / 68.0 | 35, 88 | p | 27.1 M | 510 k | 81.4 | 22.2 |
| 16 | RT sequence context dependency | 2 oligo. | ligate | 0.37 M | 42.7 / 15.1 | 151 | p | (500 k / 400 k) | 2.9 k | (44.1 / 60.5) | (39.1 / 27.2)) |
| 17 | Signature vs. occupancy | oligo. | in vitro transcr. | 1.77 M | 89.1 / 81.1 | 35, 88 | p | 16.0 M | 350 k | 8.2 | 3.1 |
| 18 | Signature vs. occupancy | oligo. | synthetic | 2.00 M | 90.1 / 83.1 | 35, 88 | p | 21.2 M | 480 k | 41.5 | 11.7 |
| 19 | Signature vs. occupancy | oligo. | synthetic | 1.72 M | 91.0 / 84.4 | 35, 88 | p | 20.0 M | 450 k | 48.5 | 12.8 |
| 20 | Signature vs. occupancy | oligo. | synthetic | 2.17 M | 91.8 / 86.0 | 35, 88 | p | 26.4 M | 610 k | 64.0 | 25.0 |
| S21 | Positive control | total tRNA | *S. cerevisiae* | 1.95 M | 44.4 / 49.7 | 80, 80 | p | 145 k | 5.6 k | 51.9 | 60.2 |
| S22 | m^1^A_58_ knockout | total RNA | *S. cerevisiae* | 3.21 M | 58.8 / 51.9 | 80, 80 | p | 571 k | 11.2 k | 0.2 | 0.3 |
| S23 | Novel sites | total tRNA | *T. brucei* | 1.36 M | 4.8 / 3.9 | 80, 80 | p | 7.5 k | 0.4 k | 36.6 | 83.0 |
| S24 | Signature vs. occupancy | tRNA^Arg_1^ | *T. brucei* | 1.79 M | 13.1 / 12.4 | 80, 80 | p | 2.6 M | 85 k | 18.3 | 82.8 |


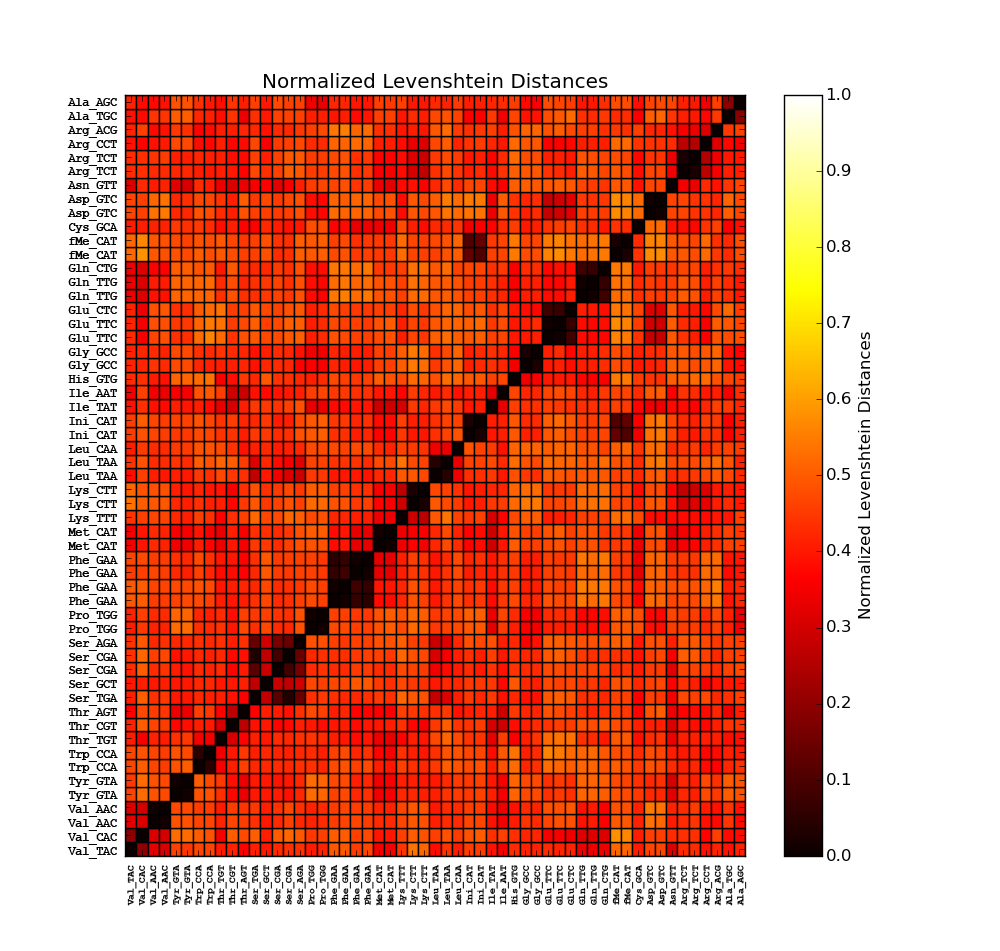


**Fig. S2: Pairwise Levenshtein edit distances of cytosolic tRNA sequences of *S. cerevisiae*.** Normalization was done by division of each distance value by the length of the longer of two compared sequences.


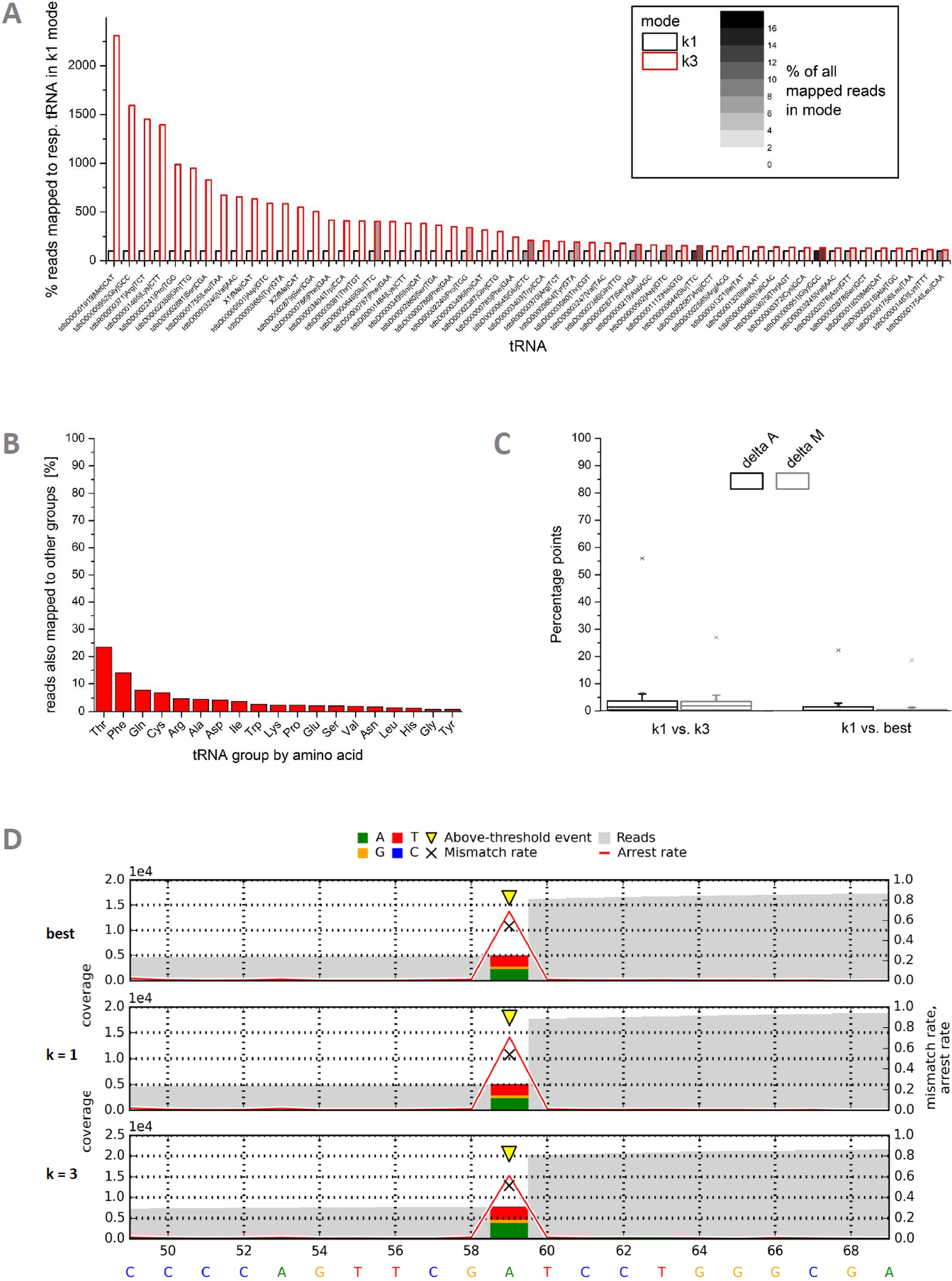


**Fig. S3: RT signature vs multiple mappings.** A) Relative read count comparison of k = 1 (“report best only”, set as 100 % for each tRNA) and k = 3 (“report best three”) modes for valid alignments by Bowtie2. B) Isotype confusion behavior for k = 3. The red bars indicate the relative amount of mapped reads per tRNA also mapped to tRNA(s) of a different acceptor group. C) Distribution of absolute difference in arrest and mismatch rates: k = 1 vs. k = 3 and k = 1 vs. “best”. D) Exemplary comparison of m^1^A_58_ (sequence position 59) RT signatures for reporting modes k = 1, k = 3 and “best” (default) in yeast’s cytosolic tRNA^Val_AAC^.


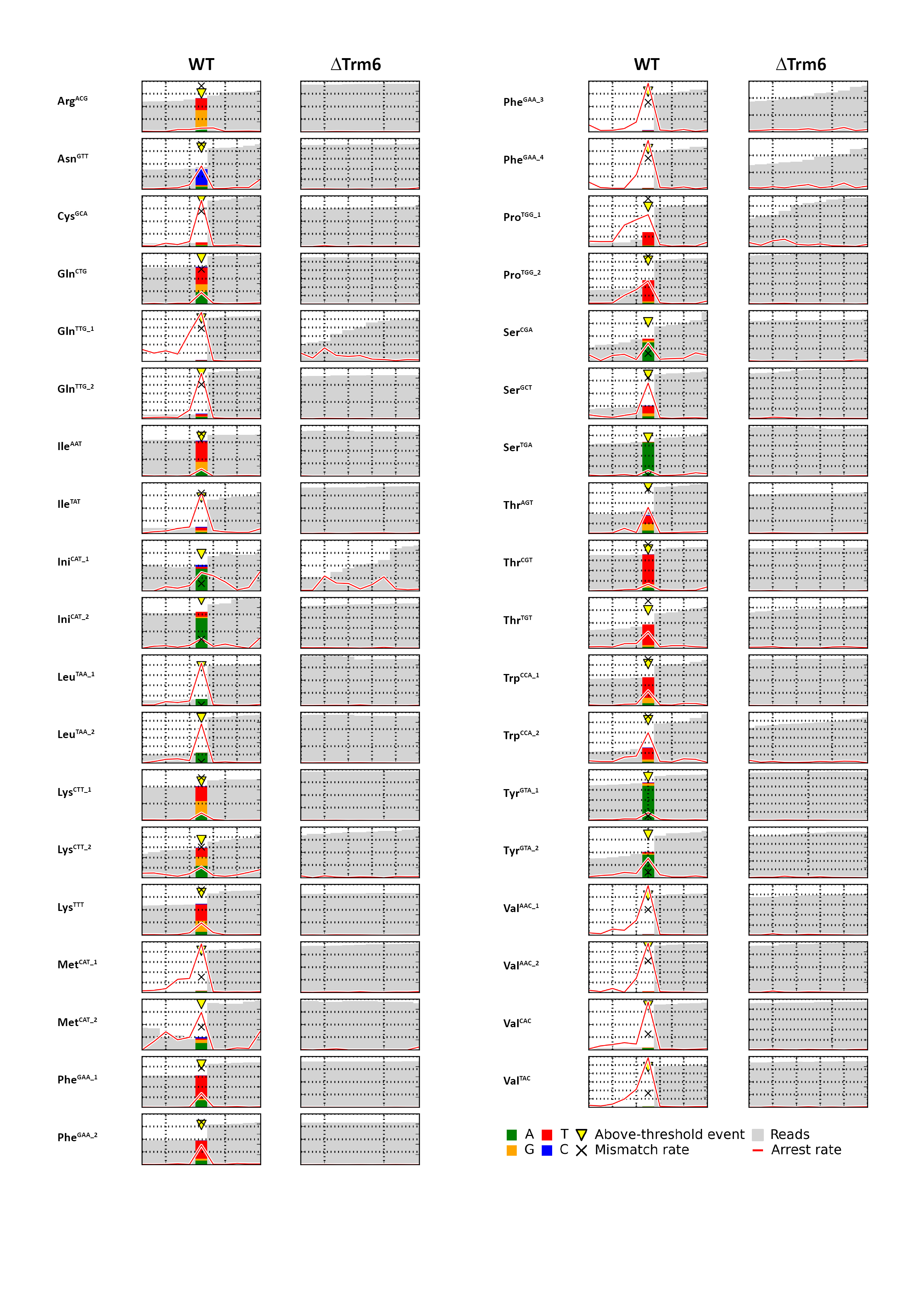


**Fig. S4: m^1^A_58_ signature compilation** from 37 cytosolic tRNAs of wildtype and m^1^A-negative (ΔTrm6) S. cerevisiae. Plot scope: 5 bp upstream and 5 bp downstream of m^1^A. Arrest and mismatch rates range on a [0, 1] scale as in the main article.


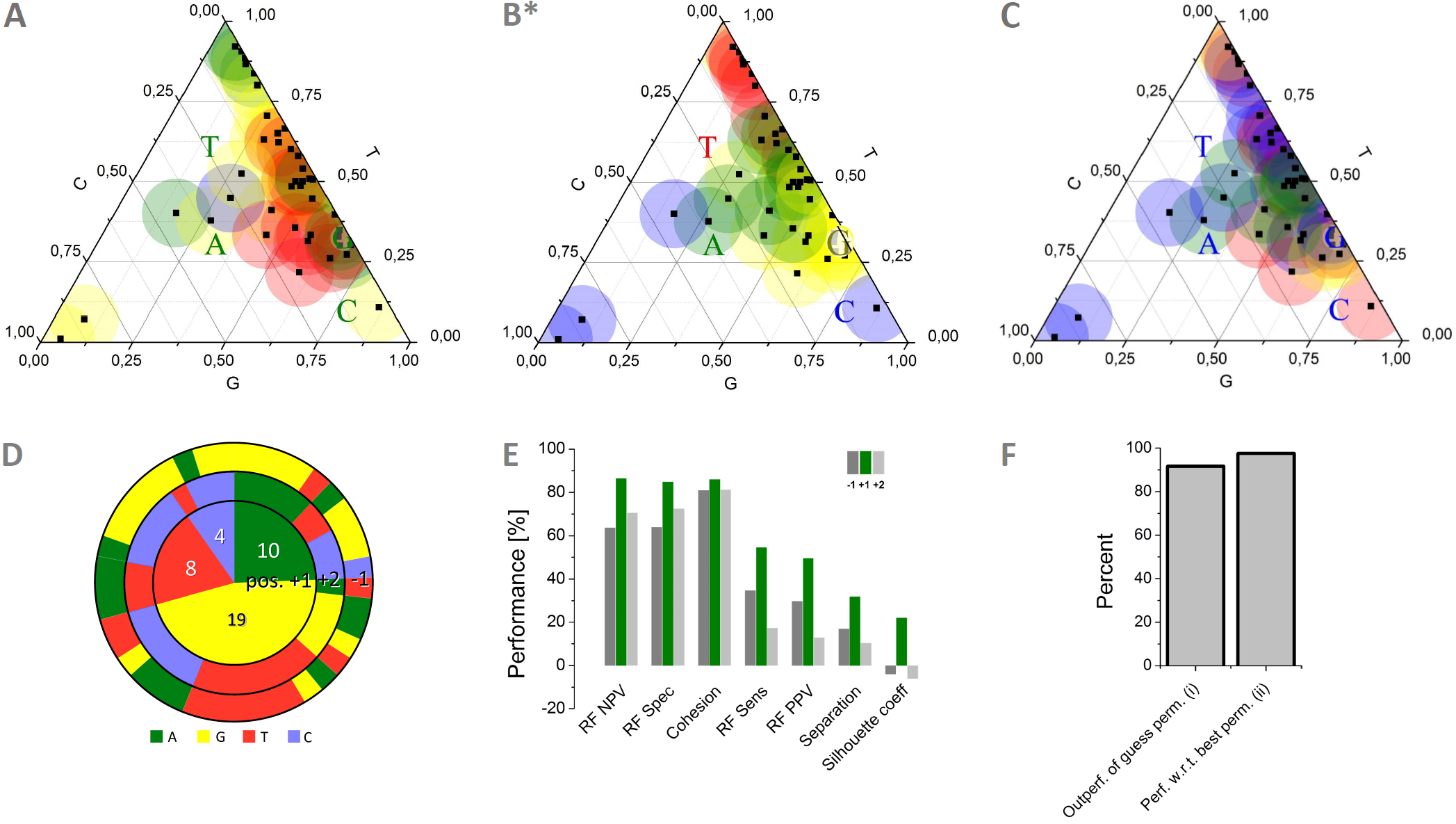


**Figure S5:** **RT sequence context.** Mismatch composition at m^1^A site by nucleotide configuration at -1 (A), +1 (B*, identical with Figure 4 A from main document) and +2 (C). Data points from revolver oligonucleotides are specified by base at +1. D) Observed combinations of base configurations at positions +1, +2 and -1 relative to m^1^A. E) Positional comparison by clustering measures cohesion, separation & silhouette coefficient as well as Random Forest prediction performance in ten repetitions of seven-fold stratified cross-validation. Means for negative and positive predictive values (NPV & PPV), sensitivity and specificity indicate the model’s performance predicting the base configuration at position -1, +1 or +2 from the m^1^A site’s mismatch composition. F) Accordance of revolver assay with m^1^A pool. Knowing that the mismatch compositions of the synthetic instances correspond to four distinct populations of the global pool, 22 of 23 alternative permutations are outperformed *(i)* by the actual assignment, based on the mean of the four corresponding distances to cluster centers (MDC). The correct assignment ranks at 97.6 % of the mean MDC of the best-performing permutation (*ii*).

**Method S1: Computational analysis of sequence context mediated mismatch composition**

Evaluation of determinism regarding mismatch compositions driven by neighboring bases was done using both, descriptive and inferential statistics. Fig. S1, E shows seven measures, comparing positions *-1*, *+1* and *+2* in what is generalized as *performance* in RT context-mediated misincorporation pattern determinism. Separation describes the average inter-cluster center distance, while cohesion is the inverse of average intra-cluster deviation from the corresponding center. Edit distances were calculated for clusters with more than one data points only. They are defined as the alteration of the two least-diverging mismatch types
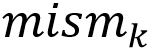
 of an arbitrary pair of misincorporation patterns
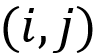
 required to transform one data point into the other, equivalent to:


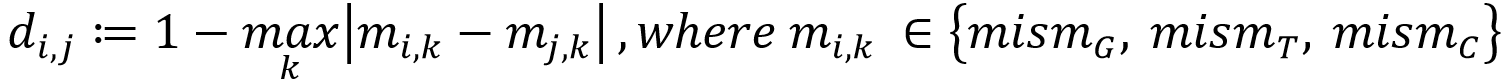


In case of cohesion, performance scale normalization was performed constituting the weakest performance as the maximum possible average deviation of data points from cluster means, amounting to 2/3 in the hypothetic worst case of uniform distribution of a cluster’s data points to the three corners of the ternary plot. Mean calculation from the four cohesion values was done using weights corresponding to the number of data points in each cluster. The maximum theoretical average inter-cluster center distance, used for normalization of the separation parameter, was set to 100 percentage points, corresponding to three perfectly condensed clusters of contrary misincorporation characteristics. Determinism analysis was supplemented by performance assessment of a Random Forest classifier (, 500 trees) inferring the base type of a corresponding neighbor position from the mismatch composition. In a four-fold stratified cross validation, we obtained sensitivities of (34.7, 54.5 and 17.3), specificities of (64, 84.8, 72.4) as well as positive and negative predictive values of (29.8, 49.5, 12.9) resp. (63.6, 86.5, 70.6) for the model in average for positions -1, +1 and +2. Fig S1, F shows how the measurements from the revolver oligonucleotides fit the clusters of the remaining data points. Distances from revolver oligonucleotide data points to cluster centers were normalized by average *intra* cluster distances, accounting for variance. Additional normalization with cluster member counts corrected for *a priori* class likelihoods.


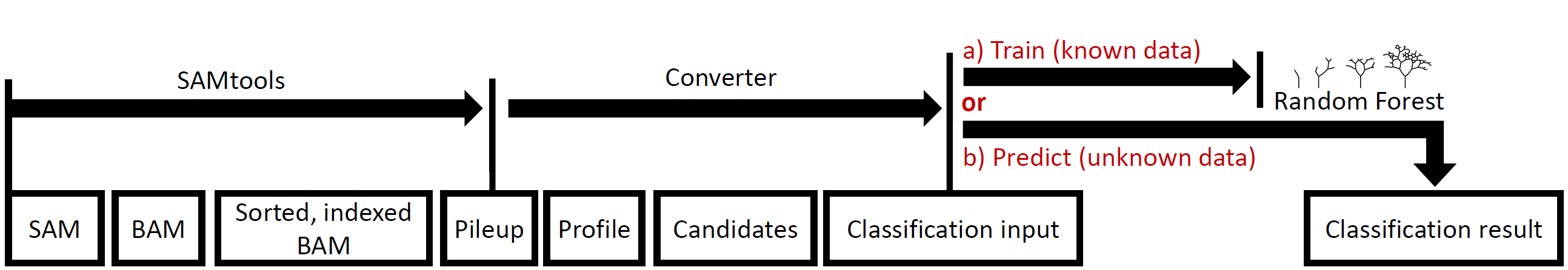


**Fig. S6: Workflow - Data processing for prediction.**

**Table S3: Profile format.**

| **ref_seg** | **pos** | **refbase** | **cov** | **matches** | **A** | **G** | **T** | **C** | **arrest** | **mismatch** |
| --- | --- | --- | --- | --- | --- | --- | --- | --- | --- | --- |
| tdbD00003245|Sacch…cer..|4932|Val|AAC | 59 | A | 7793 | 3822 | 3822 | 858 | 3057 | 56 | 0.612 | 0.515 |

**Table S4: Classification input format.** CSA denotes context sensitive arrest rate as defined in methods section.

| **arrest** | **mismatch** | **mismatch per arrest** | **CSA** | **G mism** | **T mism** | **C mism** | **mod. type** |
| --- | --- | --- | --- | --- | --- | --- | --- |
| 0.76 | 0.569 | 0.749 | 8 | 0.29 | 0.448 | 0.262 | 'm1A' |

**Table S5: Random Forest performance - 10 rep. 5-fold cross-validation.** SD^R^ is the absolute standard deviation of a mean value calculated from 10 runs. SD^F^ is the mean SD of 10x5 = 50 foldwise outcomes for the corresponding performance measure.

| **performance for class m^1^A (avg. of 10 runs)** | **low resemblance** | **high resemblance** |
| --- | --- | --- |
| sensitivity [%] (+/- SD)^R^ (+/- SD)^F^ | 96.2 (+/- 1.0)^R^ (+/- 6.2)^F^ | 88.9 (+/- 1.4) ^R^  (+/- 9.1)^F^ |
| specificity [%] (+/- SD)^R^ (+/- SD)^F^ | 96.9 (+/- 2.0) ^R^ (+/- 4.1)^F^ | 87.0 (+/- 2.8) ^R^ (+/- 10.5)^F^ |
| positive predictive value (PPV) [%] (+/- SD)^R^ (+/- SD)^F^ | 97.1 (+/- 1.8) ^R^ (+/- 3.8)^F^ | 87.4 (+/- 2.4) ^R^ (+/- 8.9)^F^ |
| negative predictive value (NPV) [%] (+/- SD)^R^ (+/- SD)^F^ | 96.6 (+/- 0.9) ^R^ (+/- 5.5)^F^ | 89.4 (+/- 1.1) ^R^ (+/- 8.1)^F^ |

**Table S6: Random Forest performance - 10 rep. leave-one-m^1^**A-out cross-validation

| **performance for class m^1^A (avg. of 10 runs)** | **low resemblance** | **high resemblance** |
| --- | --- | --- |
| sensitivity [%] +/- SD | 96.0 +/- 0.9 | 89.3 +/- 1.9 |
| specificity [%] +/- SD | 98.0 +/-1.8 | 86.7 +/- 3.8 |
| positive predictive value (PPV) [%] +/- SD | 95.1 +/-1.4 | 82.8 +/- 3.2 |
| negative predictive value (NPV) [%] +/- SD | 96.1 +/- 2.0 | 81.4 +/- 4.2 |


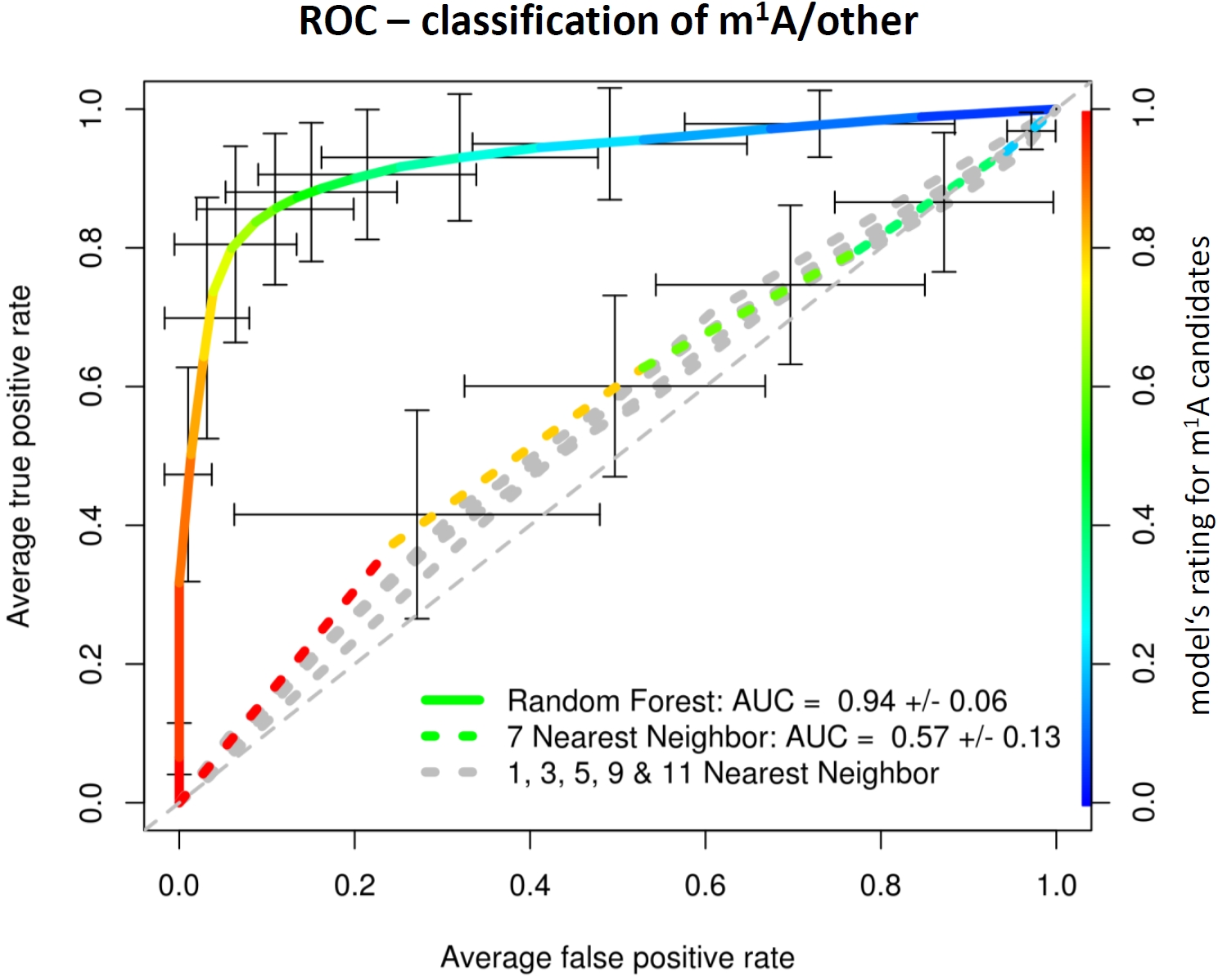


**Fig. S7: Receiver operating characteristic (ROC)** plot showing the areas under curve (AUC) for Random Forest and k Nearest Neighbor (NN) supervised prediction of m^1^A vs. other sites. The curves are averaged from 10 repetitions of a 5-fold cross validation. Error bars show the standard deviations of the ROC curve at the models’ rating scores attributed to the m^1^A candidates. The grey diagonal corresponds to the performance of a guessing classifier.


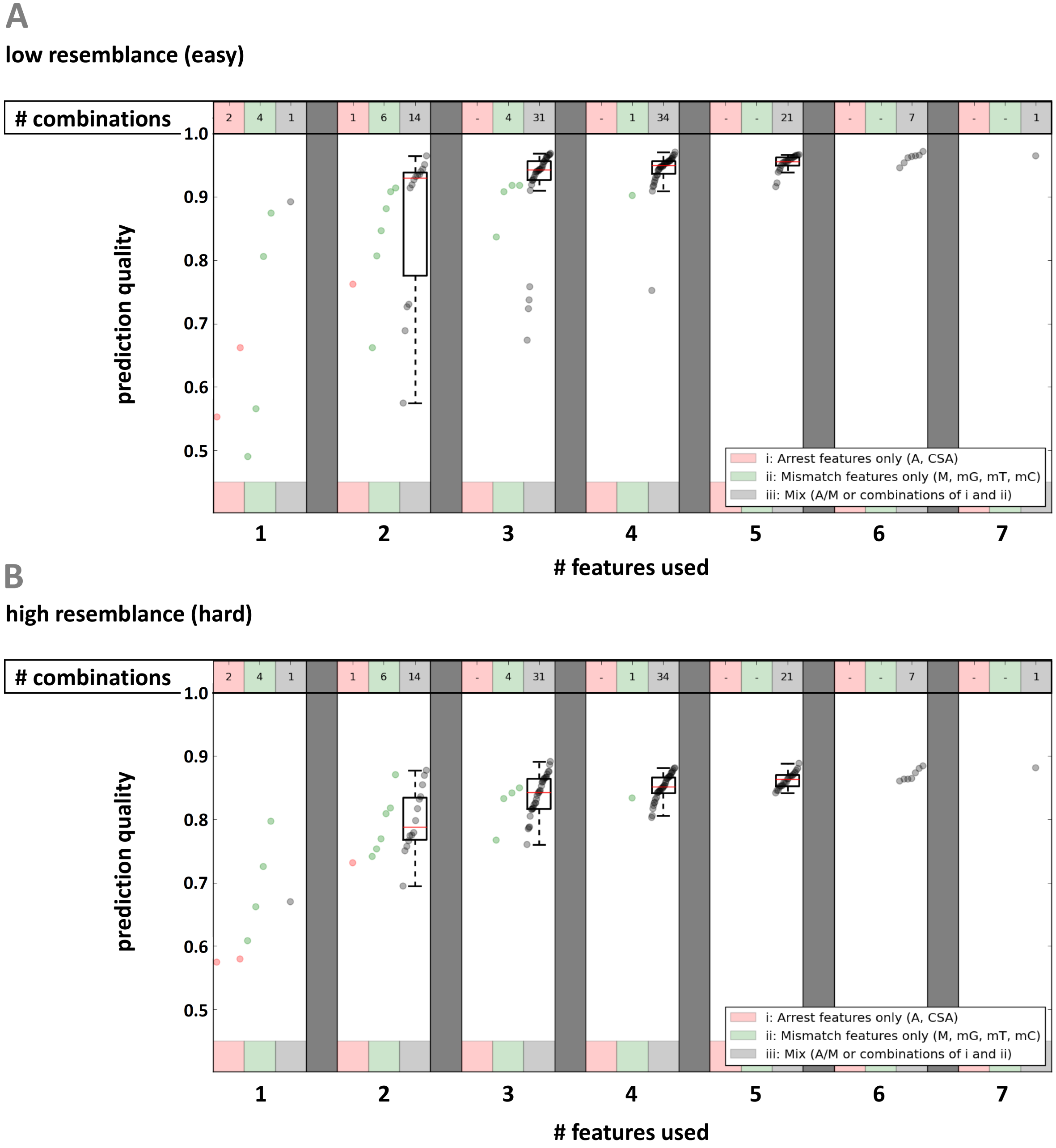


**Fig. S8: Prediction quality vs. number and category of predictors.** Quality is given as mean of sensitivity, specificity, positive and negative predictive value. A) Low and B) high mean resemblance of non-m^1^As to m^1^As as specified in subsection ‘Supervised prediction of m^1^A by machine learning’ results. 2^7^-1=127 combinations of 7 used or omitted features were tested for classification by Random Forest. The number of data points in each column corresponds to the possible combinations for the categories i)-iii) using the respective feature count 1-7.


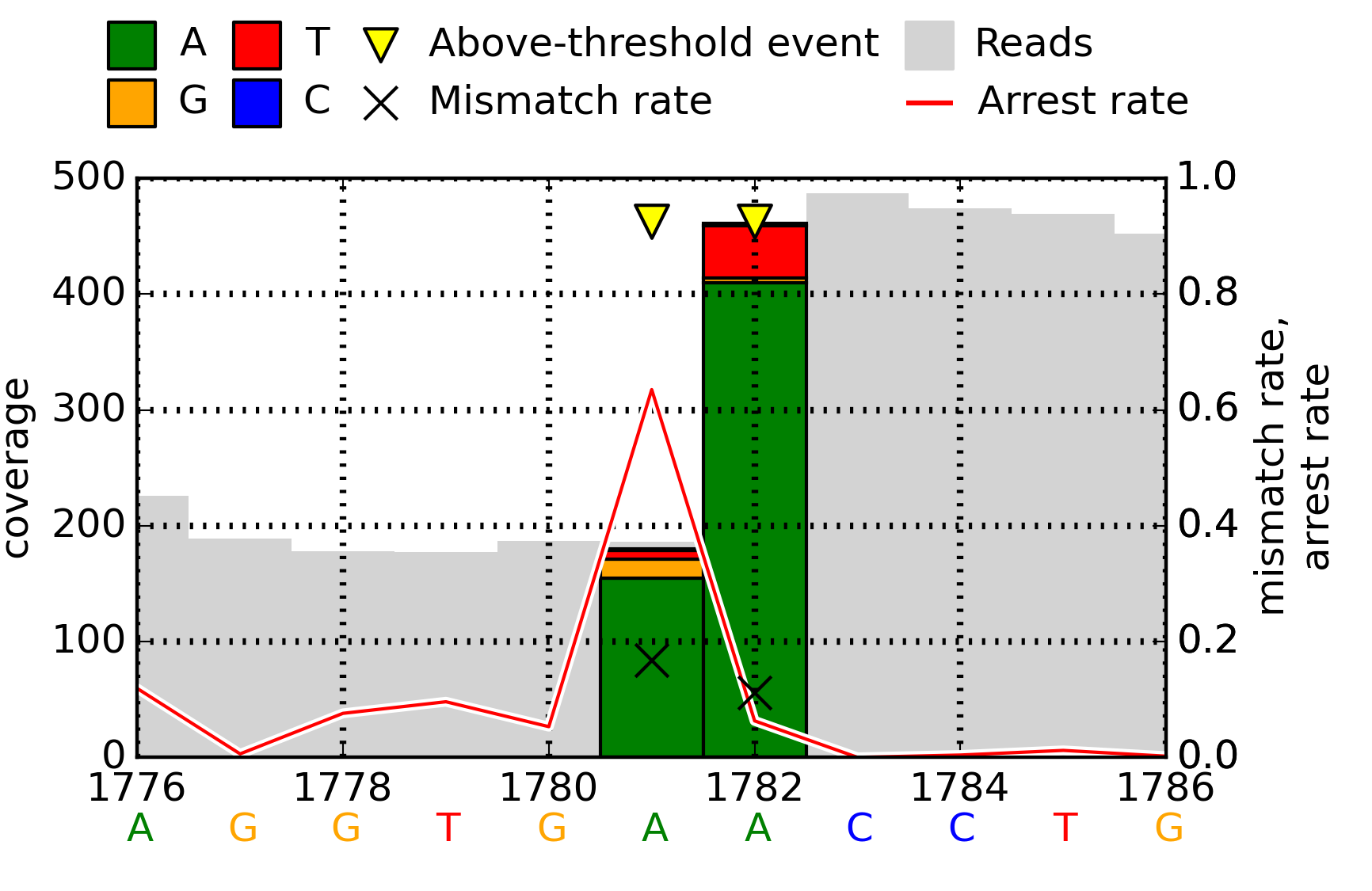


**Fig. S9: RT signatures of m^6,6^As 1781 and 1782 in yeast 18S rRNA.** For a position p, the arrest rate reflects the relative amount of mapped reads ending at p+1, i.e. not covering p.


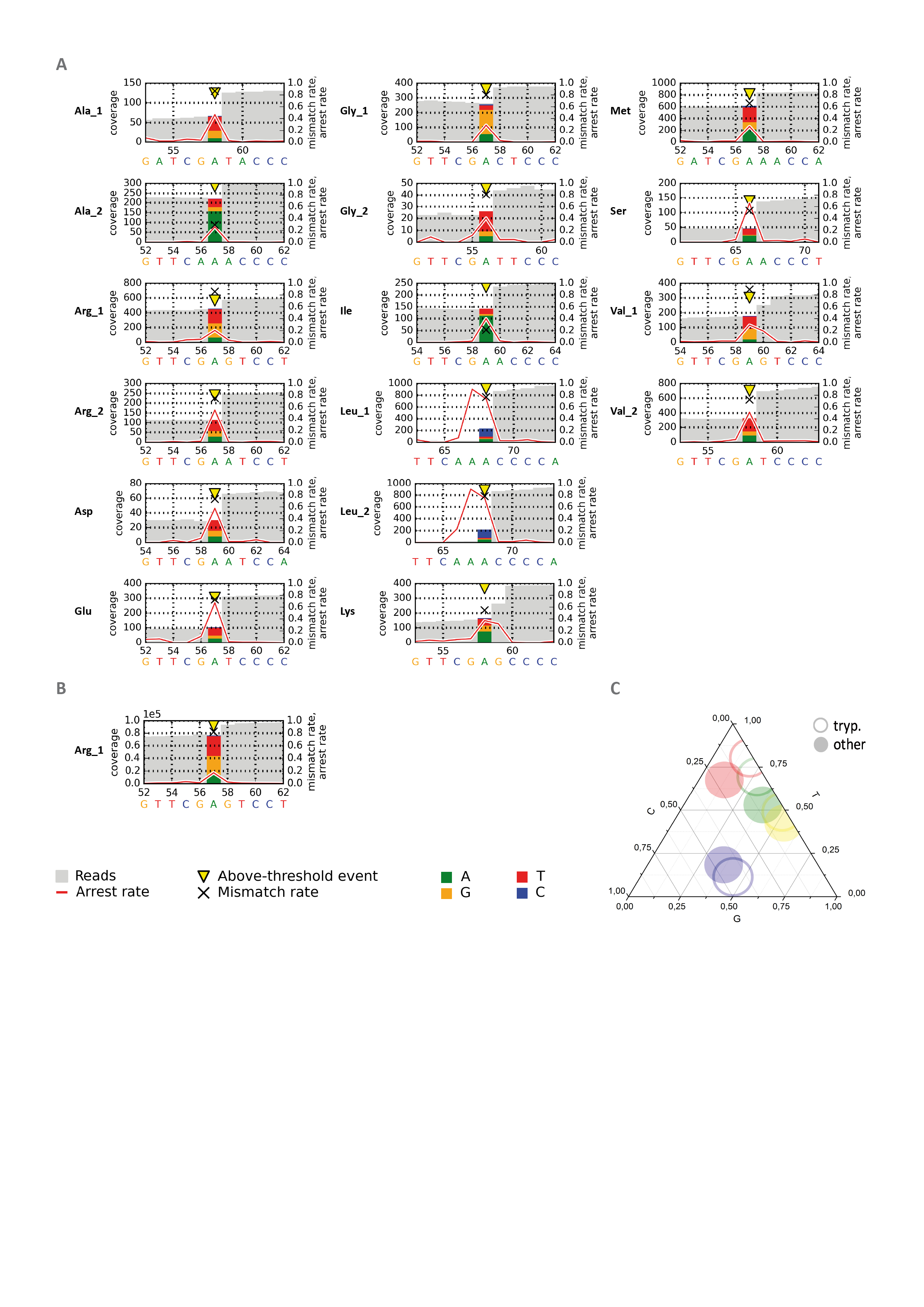


**Fig. S10: Trypanosomal m^1^A_58_.** A) 16 single tRNA profiles from total tRNA preparation from *Trypanosoma*. B) Sequencing profile of a purified sample of tRNA^Arg_UCG^. C) Mismatch composition by base configuration at pos +1. The data points taken from Figure S10A were treated in the same way as described for yeast and averaged, then visualized as open circles. For comparison, yeast data averaged from Figure 3B are plotted in closed circles.

**Table S7: LC-MS/MS quantification of m^1^A.**

| **ID** | **Sample** | **Material** | **Interest** | **m^1^A/molecule** | **% m^1^A/A** |
| --- | --- | --- | --- | --- | --- |
| 3 | *S. cer.* Δ trm6 | total tRNA | m^1^A_58_ knockout |  | 0.04 |
| 4 | *S. cer.* wt | total tRNA | positive control |  | 3.89 |
| 5 | *S. cer.* 25S wt | rRNA | wildtype | 1.50 |  |
| 6 | *S. cer.* 25S Δrrp8 | rRNA | single knockout m^1^A_645_ | 0.73 |  |
| 7 | *S. cer.* 25S Δbmt2 | rRNA | single knockout m^1^A_2142_ | 0.77 |  |
| 8 | *S. cer.* 25S Δrrp8 + Δbmt2 | rRNA | double knockout m^1^A_645_ and m^1^A_2142_ | 0.01 |  |
| 9 | *S. pactum* | total RNA | m^1^A on SSU of rRNA |  | 0.15 |
| 10 | *H. sapiens* | rRNA | Homologous identification |  | 0.15 |
| 11 | *M. musculus* | rRNA | Homologous identification |  | 0.09 |
| 12 | revolver m^1^A-G | synthetic oligo. | RT sequence context dependency | 0.77 |  |
| 13 | revolver m^1^A-C | synthetic oligo. | RT sequence context dependency | 0.82 |  |
| 14 | revolver m^1^A-U | synthetic oligo. | RT sequence context dependency | 0.92 |  |
| 15 | revolver m^1^A-A | synthetic oligo. | RT sequence context dependency | 0.79 |  |
| 17 | A-G | *in vitro* transcr. | negative control | 0.00 |  |
| S24 | Signature vs. occupancy | tRNA^Arg_UCG^ | Novel site | 1.01 |  |

References

1. Rousseeuw, P.J. (1987) Silhouettes: a Graphical Aid to the Interpretation and Validation of Cluster Analysis. *Computational and Applied Mathematics*, **20**, 53-65.

2. Liaw, A., & Wiener, M. (2002) Classification and regression by randomForest. *R news*, **2**, 18-22.

3. Sing, T., Sander, O., Beerenwinkel, N. and Lengauer, T. (2005) ROCR: visualizing classifier performance in R. *Bioinformatics*, **21**, 3940-3941.
